# Impact of Big Data Analytics on People’s Health: Overview of Systematic Reviews and Recommendations for Future Studies

**DOI:** 10.2196/27275

**Published:** 2021-04-13

**Authors:** Israel Júnior Borges do Nascimento, Milena Soriano Marcolino, Hebatullah Mohamed Abdulazeem, Ishanka Weerasekara, Natasha Azzopardi-Muscat, Marcos André Gonçalves, David Novillo-Ortiz

**Affiliations:** 1 School of Medicine Universidade Federal de Minas Gerais Belo Horizonte, Minas Gerais Brazil; 2 Department of Medicine School of Medicine Medical College of Wisconsin Wauwatosa, WI United States; 3 Department of Internal Medicine University Hospital Universidade Federal de Minas Gerais Belo Horizonte, Minas Gerais Brazil; 4 School of Medicine and Telehealth Center Universidade Federal de Minas Gerais Belo Horizonte, Minas Gerais Brazil; 5 Department of Sport and Health Sciences Technical University Munich Munich Germany; 6 School of Health Sciences Faculty of Health and Medicine The University of Newcastle Callaghan Australia; 7 Department of Physiotherapy Faculty of Allied Health Sciences University of Peradeniya Peradeniya Sri Lanka; 8 Division of Country Health Policies and Systems World Health Organization, Regional Office for Europe Copenhagen Denmark; 9 Department of Computer Science Institute of Exact Sciences Federal University of Minas Gerais Belo Horizonte, Minas Gerais Brazil

**Keywords:** public health, big data, health status, evidence-based medicine, big data analytics, secondary data analysis, machine learning, systematic review, overview, World Health Organization

## Abstract

**Background:**

Although the potential of big data analytics for health care is well recognized, evidence is lacking on its effects on public health.

**Objective:**

The aim of this study was to assess the impact of the use of big data analytics on people’s health based on the health indicators and core priorities in the World Health Organization (WHO) General Programme of Work 2019/2023 and the European Programme of Work (EPW), approved and adopted by its Member States, in addition to SARS-CoV-2–related studies. Furthermore, we sought to identify the most relevant challenges and opportunities of these tools with respect to people’s health.

**Methods:**

Six databases (MEDLINE, Embase, Cochrane Database of Systematic Reviews via Cochrane Library, Web of Science, Scopus, and Epistemonikos) were searched from the inception date to September 21, 2020. Systematic reviews assessing the effects of big data analytics on health indicators were included. Two authors independently performed screening, selection, data extraction, and quality assessment using the AMSTAR-2 (A Measurement Tool to Assess Systematic Reviews 2) checklist.

**Results:**

The literature search initially yielded 185 records, 35 of which met the inclusion criteria, involving more than 5,000,000 patients. Most of the included studies used patient data collected from electronic health records, hospital information systems, private patient databases, and imaging datasets, and involved the use of big data analytics for noncommunicable diseases. “Probability of dying from any of cardiovascular, cancer, diabetes or chronic renal disease” and “suicide mortality rate” were the most commonly assessed health indicators and core priorities within the WHO General Programme of Work 2019/2023 and the EPW 2020/2025. Big data analytics have shown moderate to high accuracy for the diagnosis and prediction of complications of diabetes mellitus as well as for the diagnosis and classification of mental disorders; prediction of suicide attempts and behaviors; and the diagnosis, treatment, and prediction of important clinical outcomes of several chronic diseases. Confidence in the results was rated as “critically low” for 25 reviews, as “low” for 7 reviews, and as “moderate” for 3 reviews. The most frequently identified challenges were establishment of a well-designed and structured data source, and a secure, transparent, and standardized database for patient data.

**Conclusions:**

Although the overall quality of included studies was limited, big data analytics has shown moderate to high accuracy for the diagnosis of certain diseases, improvement in managing chronic diseases, and support for prompt and real-time analyses of large sets of varied input data to diagnose and predict disease outcomes.

**Trial Registration:**

International Prospective Register of Systematic Reviews (PROSPERO) CRD42020214048; https://www.crd.york.ac.uk/prospero/display_record.php?RecordID=214048

## Introduction

Big data analytics tools handle complex datasets that traditional data processing systems cannot efficiently and economically store, manage, or process. Through the application of artificial intelligence (AI) algorithms and machine learning (ML), big data analytics has potential to revolutionize health care, supporting clinicians, providers, and policymakers for planning or implementing interventions [1], faster disease detection, therapeutic decision support, outcome prediction, and increased personalized medicine, resulting in lower-cost, higher-quality care with better outcomes [1,2].

In 2018, the World Health Organization (WHO) proposed the expedited 13th General Programme of Work (GPW13), which was approved and adopted by its 194 Member States, focusing on measurable impacts on people’s health at the state level to transform public health with three core features: enhanced universal health coverage, health emergencies protection, and better health and well-being [3]. Forty-six outcome target indicators emerged from the GPW13, covering a range of health issues [3]. Big data analytics may help to support health policy decision-making, accelerate the achievement of the GPW13 core priorities and targets, and guide the roadmap for the European region based on the European Programme of Work (EPW) 2020/2025 [4,5].

Therefore, the aim of this study was to provide an overview of systematic reviews that assessed the effects of the use of big data analytics on people’s health according to the WHO core features defined in the GPW13 and the EPW. We included complex reviews that assessed multiple interventions, different populations, and differing outcomes resulting from big data analytics on people’s health, and identified the challenges, opportunities, and best practices for future research.

## Methods

### Study Design

This study was designed to provide an overview of systematic reviews in accordance with guidelines from the Cochrane Handbook for Systematic Reviews of Interventions, along with the Preferred Reporting Items for Systematic Reviews and Meta-Analyses (PRISMA) and the QUOROM (Quality of Reporting of Meta-analyses) guidelines [6-8]. The study protocol is published on PROSPERO (CRD42020214048).

### Search Strategy

To identify records assessing the effect of big data analytics on people’s health, aligned with the WHO health indicators defined in the GPW13 (Textbox 1), a comprehensive and systematic search was performed using six multidisciplinary databases from their inception to September 21, 2020. The search strategy was designed in collaboration with a senior librarian and is described in detail in Multimedia Appendix 1.

References were imported into reference management software (EndNote X9) and duplicates were removed. Unique records were uploaded onto the Covidence Platform (Veritas Health Innovation) for screening, data extraction, and quality assessment. A manual search of reference lists was performed to supplement the search.

**List of 46 World Health Organization health indicators defined at the Thirteenth General Programme of Work.**

- Number of persons affected by disasters (per 100,000 population)
- Domestic general government health expenditure (% of general government expenditure)
- Prevalence of stunting in children under 5 (%)
- Prevalence of wasting in children under 5 (%)
- Prevalence of overweight in children under 5 (%)
- Maternal mortality ratio (per 100,000 live births)
- Proportion of births attended by skilled health personnel (%)
- Under 5 mortality rate (per 1000 live births)
- Neonatal mortality rate (per 1000 live births)
- New HIV infections (per 1000 uninfected population)
- Tuberculosis incidence (per 100,000 population)
- Malaria incidence (per 1000 population at risk)
- Hepatitis B incidence (measured by surface antigen [HBsAg] prevalence among children under 5 years)
- Number of people requiring interventions against neglected tropical diseases (NTDs)
- Probability of dying from any of cardiovascular disease (CVD), cancer, diabetes, chronic renal disease (CRD) (aged 30-70 years) (%)
- Suicide mortality rate (per 100,000 population)
- Coverage of treatment interventions for substance-use disorders (%)
- Total alcohol per capita consumption in adults aged >15 years (liters of pure alcohol)
- Road traffic mortality rate (per 100,000 population)
- Proportion of women (aged 15-49 years) having need for family planning satisfied with modern methods (%)
- Universal Health Coverage (UHC) Service Coverage Index
- Population with household expenditures on health >10% of total household expenditure or income (%)
- Mortality rate attributed to air pollution (per 100,000 population)
- Mortality rate attributed to exposure to unsafe water, sanitation, and hygiene (WASH) services (per 100,000 population)
- Mortality rate from unintentional poisoning (per 100,000 population)
- Prevalence of tobacco use in adults aged ≥15 years (%)
- Proportion of population covered by all vaccines included in national programs (diphtheria-tetanus-pertussis vaccine, measles-containing-vaccine second dose, pneumococcal conjugated vaccine) (%)
- Proportion of health facilities with essential medicines available and affordable on a sustainable basis (%)
- Density of health workers (doctors, nurse and midwives, pharmacists, dentists per 10,000 population)
- International Health Regulations capacity and health emergency preparedness
- Proportion of bloodstream infections due to antimicrobial-resistant organisms (%)
- Proportion of children under 5 years developmentally on track (health, learning, and psychosocial well-being) (%)
- Proportion of women (aged 15-49 years) subjected to violence by current or former intimate partner (%)
- Proportion of women (aged 15-49 years) who make their own decisions regarding sexual relations, contraceptive use, and reproductive health care (%)
- Proportion of population using safely managed drinking-water services (%)
- Proportion of population using safely managed sanitation services and hand-washing facilities (%)
- Proportion of population with primary reliance on clean fuels (%)
- Annual mean concentrations of fine particulate matter (PM2.5) in urban areas (μg/m^3^)
- Proportion of children (aged 1-17 years) experiencing physical or psychological aggression (%)
- Vaccine coverage for epidemic-prone diseases
- Proportion of vulnerable people in fragile settings provided with essential health services (%)
- Prevalence of raised blood pressure in adults aged ≥18 years
- Effective policy/regulation for industrially produced trans-fatty acids
- Prevalence of obesity (%)
- Number of cases of poliomyelitis caused by wild poliovirus
- Patterns of antibiotic consumption at the national level

### Study Selection

Peer-reviewed publications categorized as systematic reviews assessing the effects of big data analytics on any of the GPW13 and EPW health indicators and core priorities were included, regardless of language and study design. We only considered studies in which the search was performed in at least two databases, and included a description of the search strategy and the methodology used for study selection and data extraction. We only included studies that evaluated concrete relationships between the use of big data analytics and its effect on people’s lives, according to the WHO strategic priorities and indicators. Along with the 46 indicators listed in Textbox 1, we also included studies evaluating the use of big data during the COVID-19 pandemic. To identify gaps, we included reviews focusing on challenges, best practices, and short- and long-term opportunities related to big data technologies. Nonsystematic reviews, primary studies, opinions, short communications, nonscientific articles, conference abstracts, and reviews with big data inappropriately defined were excluded.

Although big data analysis is capable of handling large volumes of data, rather than focusing on the data volume/size, we focused on the process that defines big data analytics, which includes the following phases [9]: (1) data selection, (2) data preprocessing, (3) data transformation, (4) AI/expert systems, and (5) understanding/assessment. The first three phases include subtasks such as: (i) feature selection and extraction, (ii) data cleaning, and (iii) data integration from multiple sources. The included studies covered all phases of the process. Title, abstract, and full-text screening were independently performed by two reviewers using the inclusion criteria. Any disagreements were resolved by a third independent investigator.

### Data Extraction

The following data were extracted from the retrieved articles: publication information, journal name and impact factor, study characteristics, big data characteristics, outcomes, lessons and barriers for implementation, and main limitations. Data were individually extracted by team members and cross-checked for accuracy by a second investigator.

### Assessment of Methodological Quality of Included Reviews

Two researchers independently assessed the studies using the AMSTAR 2 (A Measurement Tool to Assess Systematic Reviews 2) checklist, which includes the following critical domains, assessed in 16 items: protocol registered prior to review, adequacy of literature search, justification for excluded studies, risk of bias in included studies, appropriateness of meta-analytic methods, consideration of bias risk when interpreting results, and assessing the presence and likely impact of publication bias [10]. Appropriateness to each appraisal feature was rated as yes, no, partial yes, not applicable, or unclear. Any conflict was resolved by a third party. Studies with a review protocol tracking number were analyzed. A final summary score was given to each included record, rated as “critically low,” “low,” “moderate,” or “high” [10].

### Data Synthesis

Results are reported in summary tables and through a narrative synthesis, grouping studies assessing the same disease or condition, and identifying challenges and opportunities. We also schematically represent the evidence and gaps from these reviews as an overall synthesis.

## Results

### Reviews Retrieved

**Figure 1:**
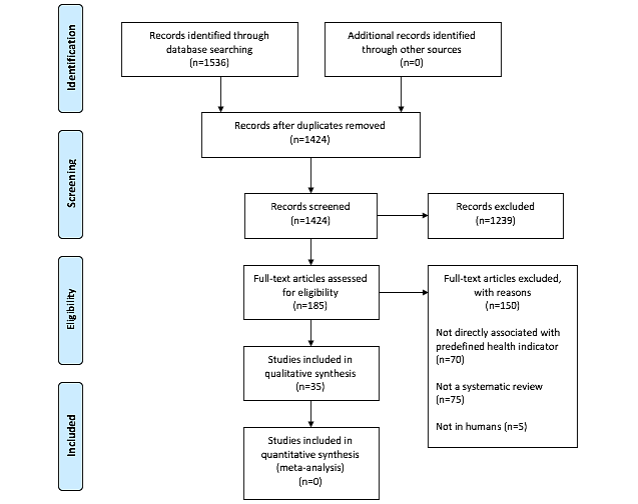
Flow chart of the different phases of article retrieval.

The search retrieved 1536 publications, 112 of which were duplicates. Most of the studies were excluded after title and abstract analysis (n=1237), leaving 185 selected for full-text screening, and 35 [11-45] were ultimately included in the final analysis after applying the eligibility criteria according to the QUOROM guidelines [8] (Figure 1). Reference list screening did not retrieve any additional review. One study under “awaiting classification” could not be retrieved.

### Quality of Evidence in Individual Systematic Reviews

Multimedia Appendix 2 shows the detailed results of the quality assessment of the 35 systematic reviews. Overall, most of the reviews (n=25) were rated with “critically low” confidence in the results using the AMSTAR 2 criteria, with 7 rated “low” and 3 rated as “moderate.” None of the reviews achieved a “high” rating. Common methodological drawbacks included omission of prospective protocol submission or publication, inappropriate search strategy, lack of independent and dual literature screening and data extraction, absence of explanation for heterogeneity among the studies, unclear or no reasons for study exclusion, and lack of risk of bias assessment.

No standard critical appraisal tools were mentioned. Among the 12 reviews that performed any quality assessment, the Quality Assessment of Diagnostic Accuracy Studies 2 tool was used in four reviews demonstrating an overall low risk of bias [14,16,27,28], whereas other tools assessed the risk of bias in studies not specifically aiming at diagnostic accuracy features. El Idrissi et al [18] used their own quality assessment tool and Luo et al [34] used an adapted version of the Critical Appraisal Skills Programme. Appraisal of the quality of evidence aligned with the Grading of Recommendations Assessment, Development and Evaluation method was reported in only one review [17]. Many reviews did not evaluate bias.

### Characteristics of Included Reviews

Summary features and main findings of the 35 systematic reviews are presented in Multimedia Appendix 3 and Multimedia Appendix 4, respectively. The included reviews were published in 34 different journals from 2007 to 2020. Most were published in English in a first-quartile journal with an impact factor ranging from 0.977 to 17.679. They covered over 2501 primary studies, involving at least 5,000,000 individuals. Only three reviews included meta-analyses, and one included a randomized clinical trial; the others were based on cohort studies.

### Data Sources and Purposes of Included Studies

Many reviews included data collected from electronic medical records, hospital information systems, or any databank that used individual patient data to create predictive models or evaluate collective patterns [12,13,16-21,24-27,30,33-35,37,38,40,42-45]. Additionally, four reviews included primary studies based on imaging datasets and databanks, assessing different parameters of accuracy [15,29,31,36]. Other reviews focused on genetic databases [28,35], data from assisted reproductive technologies [30], or publicly available data [11,14,22,32]. Four studies lacked precision about the origin of the datasets used in their analysis or did not specifically use patient data in the investigation [23,37,39,41].

The purposes of the reviews varied broadly. Generally, they (1) outlined AI applications in different medical specialties; (2) analyzed features for the detection, prediction, or diagnosis of multiple diseases or conditions; or (3) pinpointed challenges and opportunities.

### WHO Indicators and Core Priorities

Most of the studies assessed the effects of big data analytics on noncommunicable diseases [12-15,17,21,22,24,27,31,32,34,36, 38,40-44]. Furthermore, three reviews covered mental health, associated with the indicator “suicide mortality rate” [19,25,45]; three studies were related to the indicator “probability of dying from any of cardiovascular, cancer, diabetes, or chronic renal disease” [16,18,20,28,29]; and two studies were related to the indicator “proportion of bloodstream infections due to antimicrobial-resistant organisms” [26,33]. One study described technology use in disaster management and preparedness, covering the “number of persons affected by disasters” indicator [11], and one study was associated with the indicator “maternal mortality ratio” [30]. Overlap made precise classification into WHO health indicators challenging, and four studies could not be categorized because they mainly described challenges or opportunities in big data analytics [23,39] or because they were related to the COVID-19 pandemic [35,37].

### Diseases or Conditions Assessed

#### Diabetes Mellitus

AI tools associated with big data analytics in the care of patients with diabetes mellitus (DM) were assessed in six reviews that included 345 primary studies [15,20,32,38,40]. Three studies reviewed AI in screening and diagnosing type 1 or type 2 DM, providing varied ranges of accuracy, sensitivity, and specificity [20,32,40]. Variables included systolic blood pressure, body mass index, triglyceride levels, and others. Two reviews covered DM control and the clinical management of DM patients [32,40]. One noted that techniques for diabetes self-management varied among the tools evaluated and reported mean values for its robust metrics [18]. The other evaluated the use of data-driven tools for predicting blood glucose dynamics and the impact of ML and data mining [20], describing the input parameters used among data-driven analysis models. However, the authors of these reviews concluded that achieving a methodologically precise predictive model is challenging and must consider multiple parameters.

Various studies assessed the ability of big data analytics to predict individual DM complications such as hypoglycemia, nephropathy, and others [15,32,38]. Supervised ML methods, decision trees, deep neural networks, random forests (RF) learning, and support vector machine (SVM) reportedly had the best outcomes for assessing complications. One review assessed deep learning–based algorithms in screening patients for diabetic retinopathy. Of 11 studies, 8 reported sensitivity and specificity of 80.3% to 100% and 84%% to 99%, respectively; two reported accuracies of 78.7% and 81%; and one reported an area under the receiver operating curve (AUC) of 0.955 [15].

#### Mental Health

Five reviews reported on AI, data mining, and ML in psychiatry/psychology [12,14,19,25,45], most commonly assessing these techniques in the diagnosis of mental disorders. Two reviews assessed the use of ML algorithms for predicting suicidal behaviors. High levels of risk classification accuracy (typically higher than 90%) were reported in two reviews, either for adult primary care patients or teenagers [19,25]. Although the review authors stated the potential of ML techniques in daily clinical practice, limitations were highlighted, including no external validation and reporting inconsistencies.

The use of ML algorithms for early detection of psychiatric conditions was also reported [12,45]. ML was used to develop prediagnosis algorithms for constructing risk models to signal a patient’s predisposition or risk for a psychiatric/psychological health issue, for predicting a diagnosis of newly identified patients, and to differentiate mental conditions with overlapping symptomatology. For studies using structural neuroimaging to classify bipolar diseases and other diagnoses, the accuracy ranged from 52.13% to 100%, whereas studies using serum biomarkers reported an accuracy ranging from 72.5% to 77.5%.

Only one review used social media to generate analyzable data on the prevention, recognition, and support for severe mental illnesses [14]. The study included broad descriptions of ML techniques and data types for detection, diagnosis, prognosis, treatment, support, and resulting public health implications. The authors highlighted the potential for monitoring well-being, and providing an ecologically and cost-efficient evaluation of community mental health through social media and electronic records.

#### COVID-19

Two reviews reported the application of big data analytics and ML to better understand the current novel coronavirus pandemic [35,37]. One assessed data mining and ML techniques in diagnosing COVID-19 cases. Although the study did not define the best methodology to evaluate and detect potential cases, the authors noted an elevated frequency of decision tree models, naïve Bayes classifiers, and SVM algorithms used during previous pandemics.

Another review focused on SARS-CoV-2 immunization, and proposed that AI could expedite vaccine discovery through studying the virus’s capabilities, virulence, and genome using genetic databanks. That study merged discussions of deep learning–based drug screening for predicting the interaction between protein and ligands, and using imaging results linked to AI tools for detecting SARS-CoV-2 infections.

#### Oncology

Four studies described the utility of ML, computerized clinical decision systems, and deep learning in oncology [24,28,29,31]. Using computerized clinical decision support systems (DSS) significantly improves process outcomes in oncology [24]. A compelling example shows that initial decisions were modified in 31% of cases after consultation of clinical DSS, which consistently resulted in improved patient management. Furthermore, implementing clinical DSS led to an average cost reduction of US $17,000 for lung cancer patients. A remarkable workload decrease reportedly occurs when these systems are implemented in oncology facilities, leading to improved patient management and adherence to guidelines [24].

One study evaluated ML techniques in a genomic study of head and neck cancers, and found a wide range of accuracy rates (56.7% to 99.4%) based on the use of genomic data in prognostic prediction. Lastly, two studies reported accuracy levels ranging from 68% to 99.6% when using deep learning algorithms in the automatic detection of pulmonary nodules in computerized tomography images.

#### Cardiovascular and Neurological Conditions

Six studies described the effect of big data analytics in cardiology [13,16,21,42] and neurology [43,44]. One review assessed the use of ML techniques for predicting cardiac arrest [42]. Different variables were used as predictors among individual studies, including electrocardiographic parameters, heart rate variability, echocardiography, and others. Supervised ML techniques were most frequently applied to predict cardiac arrest events, with clear evidence of regression techniques and SVM algorithms. The authors reported a mean AUC of 0.76 for risk score development and efficiency evaluation [42].

Similarly, two studies assessed the use of intelligent systems in diagnosing acute coronary syndrome and heart failure [13,21], demonstrating high accuracy levels using several methods such as SVM, feature selection, and neural networks. These studies also described useful clinical features for creating prediction and diagnostic models, such as patient clinical data, electrocardiogram characteristics, and cardiac biomarkers.

Scores to identify patients at higher risk to develop QT-interval prolongation have been developed, and predictive analytics incorporated into clinical decision support tools have been tested for their ability to alert physicians of individuals who are at risk of or have QT-interval prolongation [16].

Regarding stroke, two systematic reviews evaluated using ML models for predicting outcomes and diagnosing cerebral ischemic events [43,44]. Generally, ML models were most frequently associated with mortality prediction, functional outcomes, neurological deterioration, and quality of life. The diagnosis of ischemic stroke was associated with similar or better comparative accuracy for detecting large vessel occlusion compared with humans, depending on the AI algorithm employed [44]. RF algorithms had 68% sensitivity and over 80% specificity compared with humans. Analyses of convolutional neural network (CNN) algorithms were limited, but systems using CNNs reported performance metrics on average 8% to 10% greater than those of ML employing RF, with up to 85% mean sensitivity for automatic large vessel occlusion detection. However, AI algorithm performance metrics used different standards, precluding objective comparison. Core and perfusion studies from RAPID-computed tomography and magnetic resonance imaging had the highest metrics for AI accuracy, above 80%, with some datasets showing 100% sensitivity to predict favorable perfusion mismatch. The authors noted several errors of AI use in diagnosing stroke [44].

#### Miscellaneous Conditions

Several studies reported significant improvement in disease diagnosis and event prediction using big data analytics tools, including remarkable enhancement of sepsis prediction using ML techniques [26]. Another review provided moderate evidence that ML models can reach high performance standards in detecting health care–associated infections [33].

One review focused on the diagnostic accuracy of AI systems in analyzing radiographic images for pulmonary tuberculosis, mostly referring to development instead of clinical evaluation [27]. In studies assessing accuracy, the sensitivity ranged from 56% to 97%, specificity ranged from 36% to 95%, and the AUC ranged from 78% to 99%.

One review also assessed multiple sclerosis diagnosis. Among detection methodologies, rule-based and natural language processing methods were deemed to have superior diagnostic performance based of elevated accuracy and positive predictive value [41]. This study indicates that these methods have potential impacts for early recognition of the disease, increasing quality of life, and allowing prompt pharmacological and nonpharmacological intervention.

Asthma exacerbation events and predictive models for early detection were evaluated in one review, which reached a pooled diagnostic ability of 77% (95% CI 73%-80%) [17]. Among the included studies, most models for predicting asthma development had less than 80% accuracy. None of the 42 studies modeled the reincidence of exacerbation events, and overall accuracy performance was considered inadequate. However, the authors encouraged creating models using large datasets to increase prediction accuracy levels. Logistic regression and Cox proportional hazard regression appeared to be the most commonly used methodologies. Gastric tissue disease and the usability of deep learning techniques were evaluated in one study [36]. CNN was the most common model used for gastric problem classification or detection. Additionally, residual neural network and fully convolutional network were considered to be appropriate models for disease generation, classification, and segmentation.

Two reviews analyzed the use of big data analytics and AI in public health [22,30]. One listed the impact of continuous pharmacological exposure of pregnant women, emphasizing that AI could improve popular understanding of drug effects on pregnancy, mainly through: (i) reliable clinical information disclosure, (ii) adequate scientific research design, and (iii) implementation of DSS [30]. Another review assessing the use of big data in disaster preparedness evidenced that most existing methods are qualitative, covering the response phase of the disaster chain of events [11]. The utilized tools included data originating from geographic information systems, social media interfaces, and disaster prediction modeling studies.

### Challenges and Opportunities

Two systematic reviews provided narrative evaluations of the challenges of big data analytics in health care [23,39]. Evidence from these two systematic reviews, and those from the other reviews, are summarized in Textbox 2.

**Current challenges to use big data tools for peoples’ health, and future perspectives and opportunities.**

***Current Challenges***

**1. Data structure:** issues with fragmented data and incompatible or heterogeneous data formats

**2. Data security:** problems with privacy, lack of transparency, integrity, and inherent data structure

**3. Data standardization:** concerns with limited interoperability, data obtention, mining, and sharing, along with language barriers

**4. Inaccuracy:** issues with inconsistencies, lack of precision, and data timeliness

**5. Limited awareness** of big data analytics capabilities among health managers and health care professionals

**6. Lack of evidence** of big data analytics on the impact on clinical outcomes for peoples’ health

**7. Lack of skills and training** among professionals to collect, process, or extract data

**8. Managerial issues:** ownership and government dilemma, along with data management, organizational, and financial issues

**9. Regulatory, political, and legal concerns**

**10. Expenses with data storage and transfer**

***Future Perspectives and Opportunities***

**1. To improve the decision-making process** with real-time analytics

**2. To improve patient-centric health care and to enhance personalized medicine**

**3. To support early detection of diseases and prognostic assessment** by predicting epidemics and pandemics, improving disease monitoring, implementing and tracking health behaviors, predicting patients’ vulnerabilities

**4. To improve data quality, structure, and accessibility** by enabling the improvement of rapid acquisition of large volumes and types of data, in a transparent way, and the improvement of data error detection

**5. To enable potential health care cost reduction**

**6. To improve quality of care** by improving efficient health outcomes, reducing the waste of resources, increasing productivity and performance, promoting risk reduction, and optimizing process management

**7. To provide better forms to manage population health** either through early detection of diseases or establishing ways to support health policy makers.

**8. To enhance fraud detection**

**9. To enhance health-threat detection plans by governmental entities**

**10. To support the creation of new research hypotheses**

## Discussion

This overview is the first to assess the effects of big data analytics on the prioritized WHO indicators, which offers utility for noncommunicable diseases and the ongoing COVID-19 pandemic. Although the research question focused on the impact of big data analytics on people’s health, studies assessing the impact on clinical outcomes are still scarce. Most of the reviews assessed performance values using big data tools and ML techniques, and demonstrated their applications in medical practice. Most of the reviews were associated with the GPW13 indicator “probability of dying from any cardiovascular disease, cancer, diabetes, chronic respiratory disease.” This indicator outranks others because of the incidence, prevalence, premature mortality, and economic impact of these diseases [46]. Similarly, many reviews were related to “people requiring interventions against noncommunicable diseases.” The included reviews in this study addressed many necessary health-related tasks; however, the quality of evidence was found to be low to moderate, and studies assessing the impact on clinical outcomes are notably scarce.

The low to moderate quality of evidence suggests that big data analytics has moderate to high accuracy for the (1) diagnosis and prediction of complications of DM, (2) diagnosis of mental diseases, (3) prediction of suicidal behaviors, and (4) diagnosis of chronic diseases. Most studies presented performance values, although no study assessed whether big data analytics or ML could improve the early detection of specific diseases.

Clinical research and clinical trials significantly contribute to understanding the patterns and characteristics of diseases, as well as for improving detection of acute or chronic pathologies and to guide the development of novel medical interventions [47]. However, experimental/theoretical investigations, mathematical approaches, and computer-based studies hinge on handling sample size limitations and performing data imputation [48,49]. Computer-driven analysis can easily handle missing data, examine variable mechanisms in complex systems, and employ essential tools for exploratory evaluations using voluminous input data. Big data analytics can execute an operation on/process data within microseconds after generation of the dataset, allowing for real-time follow up [50,51]. These studies and prospective applications could generate innovative knowledge and promote actionable insights; however, adapting, validating, and translating scientific data into practical medical protocols or evaluation studies is necessary.

Many systematic reviews reported simple or inappropriate evaluation measures for the task at hand. The most common metric used to evaluate the performance of a classification predictive model is accuracy, which is calculated as the proportion of correct predictions in the test set divided by the total number of predictions that were made on the test set. This metric is easy to use and to interpret, as a single number summarizes the model capability. However, accuracy values and error rate, which is simply the complement of accuracy, are not adequate for skewed or imbalanced classification tasks (ie, when the distribution of observations in the training dataset across the classes is not equal), because of the bias toward the majority class. When the distribution is slightly skewed, accuracy can still be a useful metric; however, when the distribution is severely skewed, accuracy becomes an unreliable measure of model performance.

For instance, in a binary classification task with a distribution of (95%, 5%) for the classes (eg, healthy vs sick), a “dumb classifier” that simply chooses the class “healthy” for all instances will have 95% of accuracy in this task, although the most important issue in this task would be correctly classifying the “sick” class. Precision (also called the positive predictive value), which captures the fraction of correctly classified instances among the instances predicted for a given class (eg, “sick”); recall or sensitivity, which captures the fraction of instances of a class (eg, “sick”) that were correctly classified; and F-measure, the harmonic mean of precision and recall calculated per class of interest, are more robust metrics for several practical situations. The proper choice of an evaluation metric should be carefully determined, as these indices ought to be used by regulatory bodies for screening tests and not for diagnostic reasoning [52]. The most important issue is to choose the appropriate (most robust) performance metric given the particularities of each case.

Another pitfall identified among the included reviews was the lack of reporting the precise experimental protocols used for testing ML algorithms and the specific type of replication performed.

There is no formal tool for assessing quality and risk of bias in big data studies. This is an area that is ripe for development. In Textbox 3, we summarize our recommendations for systematic reviews on the application of big data and ML for people’s health based on our experience, the findings of this systematic review, and inspired by Cunha et al [53].

High variability in the results was evident across different ML techniques and approaches among the 35 reviews, even for those assessing the same disease or condition. Indeed, designing big data analysis and ML experiments involves elevated model complexity and commonly requires testing of several modeling algorithms [54]. The diversity of big data tools and ML algorithms requires proper standardization of protocols and comparative approaches. Additionally, the process of tuning the hyperparameters of the algorithms is not uniformly reported. Important characteristics essential for replicability and external validation were not frequently available. Lastly, most of the studies provide little guidance to explain the results. Without knowing *how* and *why* the models achieve their results, applicability and trust of the models in real-world scenarios are severely compromised. Therefore, we urge the testing and assessment of supervised, unsupervised, and semisupervised methodologies, with explanation and interpretation to justify the results. Moreover, we encourage hyperparameter optimization to achieve adjusted improvement of models, enhance model generalizations for untrained data, and avoid overfitting to increase predictive accuracy.

Only two published systematic reviews evaluated the impact of big data analytics on the COVID-19 pandemic. Primary studies on COVID-19 are lacking, which indicates an opportunity to apply big data and ML to this and future epidemics/pandemics [35,37]. As of November 30, 2020, many published protocols were retrieved through a standard search on PROSPERO. The titles of these review protocols showed an intention to evaluate ML tools in diagnosis and prediction, the impact of telemedicine using ML techniques, and the use of AI-based disease surveillance [55].

Although DSS are an important application of big data analytics and may benefit patient care [56-58], only two reviews assessed such systems [16,24]. One focused on predictive analytics for identifying patients at risk of drug-induced QTc interval prolongation, discussing the efficacy of a DSS that has shown evidence of reduced prescriptions for QT interval–prolonging drugs. Similarly, one study exploring the impact of DSS on quality care in oncology showed that implementing these systems might positively impact physician-prescribing behaviors, health care costs, and clinician workload.

This overview of systematic reviews updates the available evidence from multiple primary studies intersecting computer science, engineering, medicine, and public health. We used a comprehensive search strategy (performed by an information specialist) with a predefined published protocol, precise inclusion criteria, rigorous data extraction, and quality assessment of retrieved records. We avoided reporting bias through the dual and blinded examination of systematic reviews and by having one review author standardizing the extracted data.

**Recommendations for systematic reviews on the application of big data and machine learning for people’s health.**

- *Choose an appropriate evaluation measure for the task and data characteristics, and justify your choice*

Different evaluation measures such as accuracy, area under the receiver operating characteristic curve, precision, recall, and F-measure capture different aspects of the task and are influenced by data characteristics such as skewness (ie, imbalance), sampling bias, etc. Choose your measures wisely and justify your choice based on the aforementioned aspects of the task and the data.

- *Ensure the employment of appropriate experimental protocols/design to guarantee generalization of the results*

Authors should use experimental protocols based on cross-validation or multiple training/validation/test splits of the employed datasets with more than one repetition of the experimental procedure.  The objective of this criterion is to analyze whether the study assesses the capacity of generalization of each method compared in the experiments. The use of a single default split of the input dataset with only one training/test split does not fit this requirement. Repetitions are essential to demonstrate the generalization of the investigated methods for multiple training and test sets, and to avoid any suspicion of a “lucky” (single) partition that favors the authors’ method.

- *Properly tune, and explicitly report the tuning process and values of the hyperparameters of all compared methods*

The effectiveness of big data solutions and machine-learning methods is highly affected by the choice of the parameters of these methods (ie, parameter tuning). The wrong or improper choice of parameters may make a highly effective method exhibit very poor behavior in a given task. Ideally, the parameters should be chosen for each specific task and dataset using a partition of the training set (ie, validation), which is different from the dataset used to train and to test the model. This procedure is known as cross-validation on the training set or nested cross-validation.

Even if the tuning of all methods is properly executed, this should be explicitly reported in the paper, with the exact values (or range of values) used for each parameter and the best choices used. When the tuning information is missing or absent, it is impossible to determine whether the methods have been implemented appropriately and if they have achieved their maximum potential in a given task. It is also impossible to assess whether the comparison is fair, as some methods may have been used at their maximum capacity and others not.

- *Pay attention to the appropriate statistical tests*

Authors should employ statistical significance tests to contrast the compared strategies in their experimental evaluation. Statistical tests are essential to assess whether the performance of the analyzed methods in the sample (ie, the considered datasets) is likely to reflect, with certain confidence, their actual performance in the whole population. As such, they are key to support any claim of superiority of a particular method over others. Without such tests, the relative performance observed in the sample cannot, by any means, be extrapolated to the population. The choice of the tests should also reflect the characteristics of the data (ie, determining whether the data follow a normal distribution).

- *Make the data and code freely available with proper documentation*

One of the issues that hampers reproducibility of studies, and therefore scientific progress, is the lack of original implementation (with proper documentation) of the methods and techniques, and the unavailability of the original data used to test the methods. Therefore, it is important to make all data, models, code, documentation, and other digital artifacts used in the research available for others to reuse. The artifacts made available must be sufficient to ensure that published results can be accurately reproduced.

- *Report other dimensions of the problem such as model costs (time) and potential for explainability*

Effectiveness of the solutions, as captured by accuracy-oriented measures, is not the only dimension that should be evaluated. Indeed, if the effectiveness of the studied models is similar and sufficient for a given health-related application, other dimensions such as time efficiency (or the costs) to train and deploy (test) the models are essential to evaluate the practical applicability of such solutions. Another dimension that may influence the decision for the practical use of a big data or a machine-learning method in a real practical situation is the ability to understand why the model has produced certain outputs (ie, explainability). Solutions such as those based on neural networks may be highly effective when presented with huge amounts of data, but their training and deployment costs as well as their opaqueness may not make them the best choice for a given health-related application.

However, limitations exist. The inferior quality scores based on the AMSTAR 2 tool might reflect incomplete reporting and lack of adherence to substandardized review methods. There is neither an established bias risk tool specifically for big data or ML studies nor any systematic way of presenting the findings of such studies. Furthermore, most studies provided a narrative description of results, requiring summarization. Nevertheless, all of the reviews were inspected by most authors, and the most relevant data were condensed in the text or in descriptive tables.

Big data analytics provide public health and health care with powerful instruments to gather and analyze large volumes of heterogeneous data. Although research in this field has been growing exponentially in the last decade, the overall quality of evidence is found to be low to moderate. High variability of results was observed across different ML techniques and approaches, even for the same disease or condition. The diversity of big data tools and ML algorithms require proper standardization of protocols and comparative approaches, and the process of tuning the hyperparameters of the algorithms is not uniformly reported. Important characteristics essential for replicability and external validation were not frequently available.

Additionally, the included reviews in this systematic review addressed different health-related tasks; however, studies assessing the impact on clinical outcomes remain scarce. Thus, evidence of applicability in daily medical practice is still needed. Further studies should focus on how big data analytics impact clinical outcomes and on creating proper methodological guidelines for reporting big data/ML studies, as well as using robust performance metrics to assess accuracy.

## Appendix

Multimedia Appendix 1 Search strategy used in the research.

Multimedia Appendix 2 Quality assessment judgment using the AMSTAR 2 tool.

Multimedia Appendix 3 Main characteristics of included studies.

Multimedia Appendix 4 Results and limitations of included systematic reviews.

## Abbreviations

AI: artificial intelligence
AUC: area under the receiver operating characteristic curve
AMSTAR 2: A Measurement Tool to Assess Systematic Reviews 2
CNN: convolutional neural network
DM: diabetes mellitus
DSS: decision support system
EPW: European Programme of Work
GPW13: Thirteenth General Programme of Work
ML: machine learning
PRISMA: Preferred Reporting Items for Systematic Reviews and Meta-analyses
QUOROM: Quality of Reporting of Meta-analyses
RF: random forest
SVM: support vector machine
WHO: World Health Organization
