# Supplementary material for: Impact of Big Data Analytics on People’s Health: Overview of Systematic Reviews and Recommendations for Future Studies
Source: J Med Internet Res. 2021 Apr 13;23(4):e27275. doi: 10.2196/27275 (PMC8080139; doi:10.2196/27275)
Supplement: Multimedia Appendix 1 [file jmir_v23i4e27275_app1.docx]

# **Supplementary Appendix 1 – Search strategy used in the research**

# **PubMed**

1. "Big Data"[MeSH Terms] OR “Data Mining”[MeSH Terms] OR “Machine Learning”[MeSH Terms] OR “Big Data”[Title/Abstract] OR “Machine Learning”[Title/Abstract] OR “Text Mining”[Title/Abstract] OR “Textmining”[Title/Abstract] OR “Data Mining”[Title/Abstract] OR “Predictive Analytics”[Title/Abstract] OR “Data Analytics”[Title/Abstract]
2. “Universal Health Insurance”[Mesh] OR “Universal Health coverage”[Title/Abstract] OR “Service Coverage”[Title/Abstract] OR "Essential Health Service*[Title/Abstract] OR "Hospital Access"[Title/Abstract] OR "Hospital Bed*"[Title/Abstract] OR "Reproductive Health"[Mesh] OR “Maternal Health”[Mesh] OR "Infant Health"[Mesh] OR "Family Planning Services"[Mesh] OR "Modern Contraceptive"[Title/Abstract] OR "Amenorrhea"[Mesh] OR "Child Health"[Mesh] OR “Prenatal Care”[Mesh] OR "Pregnancy"[Mesh] OR “Child Immunization”[Title/Abstract] OR “Diphteria-tetanus-pertussis”[Title/Abstract] OR “DTP3”[Title/Abstract] OR “MCV2”[Title/Abstract] OR “PC3V3”[Title/Abstract] OR "Immunization"[Mesh] OR "Vaccination"[Mesh] OR "Communicable Diseases"[Mesh] OR “Tuberculosis”[Mesh] OR “Malaria”[Mesh] OR “HIV”[Mesh] OR "Noncommunicable Diseases"[Mesh] OR "NCD"[Title/Abstract] OR “Water Sanitization”[Title/Abstract] OR "Hygiene"[Mesh] OR "Cardiovascular Diseases"[Mesh] OR "Diabetes Mellitus"[Mesh] OR "Hypertension"[Mesh] OR "Blood Pressure"[Mesh] OR "Tobacco"[Mesh] OR "Pulmonary Disease, Chronic Obstructive"[Mesh] OR "Respiratory Tract Diseases"[Mesh] OR “Asthma”[Mesh] OR “Hyperlipidemias”[Mesh] OR “Hyperglycemia”[Mesh] OR "Neoplasms"[Mesh] OR "Hospital Bed Capacity"[Mesh] OR “Health Worker”[Title/Abstract] OR “Health Professional”[Title/Abstract] OR “Health Security”[Title/Abstract] OR "International Health Regulations"[Mesh] OR "ITR"[Title/Abstract] OR "Disaster Planning"[Mesh] OR "Civil Defense"[Mesh] OR “Emergency Preparedness”[Mesh] OR “Health Emergency”[Title/Abstract] OR “Emergency Prevent”[Title/Abstract] OR “Emergency Vaccine”[Title/Abstract] OR “Notifiable Event*”[Title/Abstract] OR “Emergency Detect”[Title/Abstract] OR “Emergency Respond”[Title/Abstract] OR "Emergency Medical Services"[Mesh] OR OR “Childhood Stunting”[Title/Abstract] OR “Childhood Wasting”[Title/Abstract] OR “Childhood overweight”[Title/Abstract] OR "Vaccination Coverage"[Mesh] OR "Epidemics"[Mesh] OR "Pandemics"[Mesh] OR "Health Status"[Mesh] OR "Pediatric Obesity"[Mesh] OR "Suicide"[Mesh] OR "Alcohol-Induced Disorders"[Mesh] OR “Road death*”[Title/Abstract] OR “Road Safety”[Title/Abstract] OR "Mental Health"[Mesh] OR "Tobacco Use"[Mesh] OR "Intimate Partner Violence"[Mesh] OR "Gender-Based Violence"[Mesh] OR "Sanitation"[Mesh] OR "Obesity"[Mesh] OR "Overweight"[Mesh] OR "Exercise"[Mesh] OR “Sanitation Service*”[Title/Abstract] OR "Diet"[Mesh]
3. “Systematic Review”[Title/Abstract] OR “Systematic Review"[Publication Type]

Final search algorithm

(# 1 AND # 2) AND # 3

# **Embase (Elsevier)**

1. 'big data'/exp OR 'big data' OR (big AND data) OR 'data mining'/exp OR 'machine learning'/exp OR 'machine learning' OR (('machine'/exp OR machine) AND ('learning'/exp OR learning)) OR 'text mining'/exp OR 'text mining' OR (text AND ('mining'/exp OR mining)) OR textmining OR 'data mining'/exp OR 'data mining' OR (data AND ('mining'/exp OR mining)) OR 'datamining'/exp OR datamining
2. 'universal health insurance'/exp OR (universal AND health AND coverage) OR (service AND coverage) OR (Essential AND Health AND Service) OR (Hospital AND Access) OR 'hospital bed'/exp OR 'reproductive health'/exp OR 'maternal welfare'/exp OR 'child health'/exp OR 'family planning'/exp OR (Modern AND Contraceptive) OR 'amenorrhea'/exp OR 'prenatal care'/exp OR 'pregnancy'/exp OR (Child AND Immunization) OR (Diphteria-tetanus-pertussis) OR (DTP3) OR (MCV2) OR (PC3V3) OR 'immunization'/exp OR 'vaccination'/exp OR 'communicable disease'/exp OR 'tuberculosis'/exp OR 'malaria'/exp OR 'Human immunodeficiency virus'/exp OR 'non communicable disease'/exp OR (NCD) OR (Water AND Sanitization) OR 'hygiene'/exp OR 'cardiovascular disease'/exp OR 'diabetes mellitus'/exp OR 'hypertension'/exp OR 'blood pressure'/exp OR 'tobacco'/exp OR 'chronic obstructive lung disease'/exp OR 'respiratory tract disease'/exp OR 'asthma'/exp OR 'hyperlipidemia'/exp OR 'hyperglycemia'/exp OR 'neoplasm'/exp OR 'hospital bed capacity'/exp OR 'health care personnel'/exp OR 'health security'/exp OR 'international health regulation'/exp OR (ITR) OR 'disaster planning'/exp OR 'civil defense'/exp OR (Emergency AND Preparedness) OR 'emergency health service'/exp OR (Emergency AND Prevent) OR (Emergency AND Vaccine) OR (Notifiable AND Event*) OR (Emergency AND Detect) OR (Emergency AND Respond) OR (Childhood AND Stunting) OR (Childhood AND Wasting) OR (Childhood AND overweight) OR 'vaccination coverage'/exp OR 'epidemic'/exp OR 'pandemic'/exp OR 'health status'/exp OR 'childhood obesity'/exp OR 'suicide'/exp OR 'alcoholism'/exp OR (Road AND death*) OR (Road AND Safety) OR 'mental health'/exp OR 'tobacco use'/exp OR 'partner violence'/exp OR 'gender based violence'/exp OR 'sanitation'/exp OR 'obesity'/exp OR 'exercise'/exp OR (Sanitation AND Service*) OR 'diet'/exp
3. 'systematic review'/de
4. AND [embase]/lim NOT ([embase]/lim AND [medline]/lim)

Final search algorithm

(# 1 AND # 2) AND # 3 AND #4

# **Cochrane Library**

1. MeSH descriptor: [Big Data] explode all trees OR MeSH descriptor: [Data Mining] explode all trees OR MeSH descriptor: [Machine Learning] explode all trees OR MeSH descriptor: [Data Science] explode all trees
2. MeSH descriptor: [Universal Health Insurance] explode all trees OR MeSH descriptor: [Reproductive Health] explode all trees OR MeSH descriptor: [Maternal Health] explode all trees OR MeSH descriptor: [Infant Health] explode all trees OR MeSH descriptor: [Family Planning Services] explode all trees OR MeSH descriptor: [Amenorrhea] explode all trees OR MeSH descriptor: [Child Health] explode all trees OR MeSH descriptor: [Prenatal Care] explode all trees OR MeSH descriptor: [Pregnancy] explode all trees OR MeSH descriptor: [Diphtheria-Tetanus-Pertussis Vaccine] explode all trees OR MeSH descriptor: [Immunization] in all MeSH products OR MeSH descriptor: [Vaccines] in all MeSH products OR MeSH descriptor: [Communicable Diseases] explode all trees OR MeSH descriptor: [Tuberculosis] explode all trees OR MeSH descriptor: [Malaria] explode all trees OR MeSH descriptor: [HIV] explode all trees OR MeSH descriptor: [Noncommunicable Diseases] explode all trees OR MeSH descriptor: [Hygiene] explode all trees OR MeSH descriptor: [Cardiovascular Diseases] explode all trees OR MeSH descriptor: [Diabetes Mellitus] explode all trees OR MeSH descriptor: [Hypertension] explode all trees OR MeSH descriptor: [Blood Pressure] explode all trees OR MeSH descriptor: [Tobacco] explode all trees OR MeSH descriptor: [Pulmonary Disease, Chronic Obstructive] explode all trees OR MeSH descriptor: [Respiratory Tract Diseases] explode all trees OR MeSH descriptor: [Asthma] explode all trees OR MeSH descriptor: [Hyperlipidemias] explode all trees OR MeSH descriptor: [Hyperglycemia] explode all trees OR MeSH descriptor: [Neoplasms] explode all trees OR MeSH descriptor: [Hospital Bed Capacity] explode all trees OR MeSH descriptor: [International Health Regulations] explode all trees OR MeSH descriptor: [Disaster Planning] explode all trees OR MeSH descriptor: [Civil Defense] explode all trees OR MeSH descriptor: [Civil Defense] explode all trees OR MeSH descriptor: [Emergency Medical Services] explode all trees OR MeSH descriptor: [Epidemics] explode all trees OR MeSH descriptor: [Pandemics] explode all trees OR MeSH descriptor: [Health Status] explode all trees OR MeSH descriptor: [Pediatric Obesity] explode all trees OR MeSH descriptor: [Suicide] explode all trees OR MeSH descriptor: [Alcohol-Induced Disorders] explode all trees OR MeSH descriptor: [Mental Health] explode all trees OR MeSH descriptor: [Tobacco Use] explode all trees OR MeSH descriptor: [Intimate Partner Violence] explode all trees OR MeSH descriptor: [Gender-Based Violence] explode all trees OR MeSH descriptor: [Sanitation] explode all trees OR MeSH descriptor: [Obesity] explode all trees OR MeSH descriptor: [Overweight] explode all trees OR MeSH descriptor: [Exercise] explode all trees OR MeSH descriptor: [Diet] explode all trees
3. [Cochrane Reviews selected]

Final search algorithm

(# 1 AND # 2) AND # 3

**Scopus (Elsevier)**

- - - 1. ( TITLE-ABS-KEY ( ( big AND data OR machine AND learning OR text AND mining OR examining OR data AND mining ) )
      2. TITLE-ABS-KEY ( ( Universal AND Health AND Insurance OR Universal AND Health AND coverage OR Service AND Coverage OR Essential AND Health AND Service* OR Hospital AND Access OR Hospital AND Bed* OR Reproductive AND Health OR Maternal AND Health OR Infant AND Health OR Family AND Planning AND Services OR Modern AND Contraceptive OR Amenorrhea OR Child AND Health OR Prenatal AND Care OR Pregnancy OR Child AND Immunization OR Diphteria-tetanus-pertussis OR DTP3 OR MCV2 OR PC3V3 OR Immunization OR Vaccination OR Communicable AND Diseases OR Tuberculosis OR Malaria OR HIV OR Noncommunicable AND Diseases OR NCD OR Water AND Sanitation OR Hygiene OR Cardiovascular AND Diseases OR Diabetes AND Mellitus OR Hypertension OR Blood AND Pressure OR Tobacco OR Pulmonary AND Disease AND, Chronic AND Obstructive OR Respiratory AND Tract AND Diseases OR Asthma OR Hyperlipidemias OR Hyperglycemia OR Neoplasms OR Hospital AND Bed AND Capacity OR Health AND Worker OR Health AND Professional OR Health AND Security OR International AND Health AND Regulations OR ITR OR Disaster AND Planning OR Civil AND Defense OR Emergency AND Preparedness OR Health AND Emergency OR Emergency AND Prevent OR Emergency AND Vaccine OR Notifiable AND Event* OR Emergency AND Detect OR Emergency AND Respond OR Emergency AND Medical AND Services OR Childhood AND Stunting OR Childhood AND Wasting OR Childhood AND overweight OR Vaccination AND Coverage OR Epidemics OR Pandemics OR Health AND Status OR Pediatric AND Obesity OR Suicide OR Alcohol-Induced AND Disorders OR Road AND death* OR Road AND Safety OR Mental AND Health OR Tobacco AND Use OR Intimate AND Partner AND Violence OR Gender-Based AND Violence OR Sanitation OR Obesity OR Overweight OR Exercise OR Sanitation AND Service* OR Diet ))

3. TITLE-ABS-KEY ( "systematic review" ) )

Final search algorithm

(# 1 AND # 2) AND # 3

# **Web of Science**

1. TOPIC: ((Big data OR machine learning OR text mining OR textmining OR data mining) ) AND

2. TOPIC: ((Universal Health Insurance OR Universal Health coverage OR Service Coverage OR Essential Health Service* OR Hospital Access OR Hospital Bed* OR Reproductive Health OR Maternal Health OR Infant Health OR Family Planning Services OR Modern Contraceptive OR Amenorrhea OR Child Health OR Prenatal Care OR Pregnancy OR Child Immunization OR Diphteria-tetanus-pertussis OR DTP3 OR MCV2 OR PC3V3 OR Immunization OR Vaccination OR Communicable Diseases OR Tuberculosis OR Malaria OR HIV OR Noncommunicable Diseases OR NCD OR Water Sanitization OR Hygiene OR Cardiovascular Diseases OR Diabetes Mellitus OR Hypertension OR Blood Pressure OR Tobacco OR Pulmonary Disease, Chronic Obstructive OR Respiratory Tract Diseases OR Asthma OR Hyperlipidemias OR Hyperglycemia OR Neoplasms OR Hospital Bed Capacity OR Health Worker OR Health Professional OR Health Security OR International Health RegulationsOR ITR OR Disaster Planning OR Civil Defense OR Emergency Preparedness OR Health Emergency OR Emergency Prevent OR Emergency Vaccine OR Notifiable Event* OR Emergency Detect OR Emergency Respond OR Emergency Medical Services OR Childhood Stunting OR Childhood Wasting OR Childhood overweight OR Vaccination Coverage OR Epidemics OR Pandemics OR Health Status OR Pediatric Obesity OR Suicide OR Alcohol-Induced Disorders OR Road death* OR Road Safety OR Mental Health OR Tobacco Use OR Intimate Partner Violence OR Gender-Based Violence OR Sanitation OR Obesity OR Overweight OR Exercise OR Sanitation Service* OR Diet))

3. TOPIC: ("systematic review")

Final search algorithm

(# 1 AND # 2) AND # 3

**Epistemonikos**

(Big data OR machine learning OR text mining OR textmining OR data mining) AND (Universal Health Insurance OR Universal Health coverage OR Service Coverage OR Essential Health Service* OR Hospital Access OR Hospital Bed* OR Reproductive Health OR Maternal Health OR Infant Health OR Family Planning Services OR Modern Contraceptive OR Amenorrhea OR Child Health OR Prenatal Care OR Pregnancy OR Child Immunization OR Diphteria-tetanus-pertussis OR DTP3 OR MCV2 OR PC3V3 OR Immunization OR Vaccination OR Communicable Diseases OR Tuberculosis OR Malaria OR HIV OR Noncommunicable Diseases OR NCD OR Water Sanitization OR Hygiene OR Cardiovascular Diseases OR Diabetes Mellitus OR Hypertension OR Blood Pressure OR Tobacco OR Pulmonary Disease, Chronic Obstructive OR Respiratory Tract Diseases OR Asthma OR Hyperlipidemias OR Hyperglycemia OR Neoplasms OR Hospital Bed Capacity OR Health Worker OR Health Professional OR Health Security OR International Health RegulationsOR ITR OR Disaster Planning OR Civil Defense OR Emergency Preparedness OR Health Emergency OR Emergency Prevent OR Emergency Vaccine OR Notifiable Event* OR Emergency Detect OR Emergency Respond OR Emergency Medical Services OR Childhood Stunting OR Childhood Wasting OR Childhood overweight OR Vaccination Coverage OR Epidemics OR Pandemics OR Health Status OR Pediatric Obesity OR Suicide OR Alcohol-Induced Disorders OR Road death* OR Road Safety OR Mental Health OR Tobacco Use OR Intimate Partner Violence OR Gender-Based Violence OR Sanitation OR Obesity OR Overweight OR Exercise OR Sanitation Service* OR Diet)
