# Supplementary material for: Impact of Big Data Analytics on People’s Health: Overview of Systematic Reviews and Recommendations for Future Studies
Source: J Med Internet Res. 2021 Apr 13;23(4):e27275. doi: 10.2196/27275 (PMC8080139; doi:10.2196/27275)
Supplement: Multimedia Appendix 2 [file jmir_v23i4e27275_app2.docx]

**Multimedia Appendix 2 – Quality assessment judgment using the AMSTAR 2 tool**

| **Author, year** | **AMSTAR score** | | | | | | | | | | | | | | | | **AMSTAR score summary** | | | | **Quality of the review** |
| --- | --- | --- | --- | --- | --- | --- | --- | --- | --- | --- | --- | --- | --- | --- | --- | --- | --- | --- | --- | --- | --- |
|  | **1** | **2** | **3** | **4** | **5** | **6** | **7** | **8** | **9** | **10** | **11** | **12** | **13** | **14** | **15** | **16** | **N** | **Y** | **CA** | **NA** |  |
| Alonso et al | y | n | y | y | n | n | n | y | n | n | na | na | n | n | na | y | 8 | 4 | 0 | 3 | Critically low |
| Galetsi et al, 2019 | y | n | y | y | y | y | n | y | n | n | na | na | n | n | na | n | 7 | 6 | 0 | 3 | Critically low |
| Abhari et al, 2019 | y | n | y | y | y | y | n | y | n | n | na | na | n | n | na | y | 6 | 7 | 0 | 3 | Critically low |
| Chaki et al, 2020 | y | n | n | y | n | n | n | y | n | n | na | na | n | n | na | y | 9 | 4 | 0 | 3 | Critically low |
| Albahri et al, 2020 | y | n | y | y | y | n | n | y | n | n | na | na | n | n | na | n | 8 | 5 | 0 | 3 | Critically low |
| Arani et al, 2018 | y | n | y | y | n | n | n | y | n | n | na | na | n | n | na | y | 8 | 5 | 0 | 3 | Critically low |
| Bernert et al, 2020 | y | n | y | y | y | y | n | y | n | n | na | na | n | n | na | y | 6 | 7 | 0 | 3 | Critically low |
| Bonnett et al, 2020 | y | n | y | y | y | y | n | y | y | n | y | n | y | y | y | y | 4 | 12 | 0 | 0 | Moderate |
| Burke et al, 2018 | y | n | y | y | n | n | n | y | n | n | na | na | n | n | na | y | 8 | 5 | 0 | 3 | Critically low |
| Davidson et al, 2020 | y | n | n | y | n | n | n | n | n | n | na | na | n | n | na | n | 11 | 2 | 0 | 3 | Critically low |
| Idrissi et al, 2019 | y | y | y | n | y | y | y | y | y | n | na | na | y | n | na | y | 3 | 10 | 0 | 3 | Low |
| Fleuren et al, 2020 | y | y | y | y | y | y | n | y | y | n | y | y | y | y | n | y | 3 | 13 | 0 | 0 | Moderate |
| Freeman et al, 2019 | y | n | y | n | n | n | n | y | n | n | na | na | n | n | na | n | 10 | 3 | 0 | 3 | Critically low |
| Gonçalves et al, 2020 | y | n | y | y | y | y | n | y | n | n | na | na | n | n | na | y | 6 | 7 | 0 | 3 | Critically low |
| Harris et al. 2019 | y | y | y | y | y | y | n | y | ca | n | y | n | n | n | n | y | 6 | 9 | 1 | 0 | Low |
| Javan et al, 2018 | y | n | y | y | y | n | n | y | n | n | na | na | n | n | na | n | 8 | 5 | 0 | 3 | Critically low |
| Kannan et al, 2020 | y | n | y | y | n | n | n | ca | n | n | na | na | n | n | na | y | 8 | 4 | 1 | 3 | Critically low |
| Kavakiotis et al, 2017 | y | n | y | y | n | n | n | n | n | n | na | na | n | n | na | n | 10 | 3 | 0 | 3 | Critically low |
| Klarenbeek et al, 2020 | y | y | y | y | y | y | n | y | y | n | na | na | y | n | na | n | 4 | 9 | 0 | 3 | Low |
| Kruse et al, 2016 | y | n | n | y | y | y | n | n | n | n | na | na | n | n | na | y | 8 | 5 | 0 | 3 | Critically low |
| Li et al, 2019 | y | n | y | y | y | y | n | y | n | n | na | na | n | n | na | y | 6 | 7 | 0 | 3 | Critically low |
| Librenza-Garcia et al, 2019 | y | n | y | y | y | n | n | y | n | n | y | n | n | n | n | y | 9 | 7 | 0 | 0 | Critically low |
| Luo et al, 2015 | y | n | y | y | y | y | n | y | y | n | na | na | n | n | na | y | 5 | 8 | 0 | 3 | Low |
| Murray et al, 2020 | y | n | y | y | n | y | n | y | n | n | na | na | n | n | na | y | 7 | 6 | 0 | 3 | Critically low |
| Nielsen et al, 2018 | y | n | y | y | y | y | y | y | y | n | na | na | n | n | na | y | 4 | 9 | 0 | 3 | Low |
| Patil et al, 2019 | y | y | y | y | y | y | n | y | n | n | na | na | n | n | na | n | 6 | 7 | 0 | 3 | Critically low |
| Pehrson et al, 2019 | y | n | y | y | y | y | n | y | n | n | na | na | n | n | na | y | 6 | 7 | 0 | 3 | Critically low |
| Scardoni et al, 2020 | y | n | y | y | y | y | n | y | y | n | na | na | y | y | na | n | 4 | 9 | 0 | 3 | Moderate |
| Shatte et al, 2019 | y | n | n | y | y | n | y | y | n | n | na | na | n | n | na | y | 7 | 6 | 0 | 3 | Critically low |
| Sprockel et al, 2017 | y | n | y | y | y | y | n | y | y | n | na | na | n | n | na | y | 5 | 8 | 0 | 3 | Low |
| Muensterman et al, 2018 | n | n | n | y | n | n | y | y | n | n | na | na | n | n | na | n | 10 | 3 | 0 | 3 | Critically low |
| Tripoliti et al, 2017 | y | y | n | y | n | n | y | y | n | n | na | na | n | n | na | n | 8 | 5 | 0 | 3 | Critically low |
| Wang et al, 2020 | y | y | y | y | y | y | n | y | y | n | na | na | n | n | na | y | 4 | 9 | 0 | 3 | Low |
| Woldaregay et al, 2019 | y | y | n | y | n | n | y | y | n | n | na | na | n | n | na | n | 8 | 5 | 0 | 3 | Critically low |
| Yin et al, 2019 | n | n | y | y | y | n | y | y | n | ca | na | na | n | n | na | y | 6 | 6 | 1 | 3 | Critically low |

CA: cannot answer; NA: not applicable; N: no; y: yes.
