# Supplementary material for: Impact of Big Data Analytics on People’s Health: Overview of Systematic Reviews and Recommendations for Future Studies
Source: J Med Internet Res. 2021 Apr 13;23(4):e27275. doi: 10.2196/27275 (PMC8080139; doi:10.2196/27275)
Supplement: Multimedia Appendix 3 [file jmir_v23i4e27275_app3.docx]

| Multimedia Appendix 3. Main characteristics of included studies | | | | | | | | | | | |
| --- | --- | --- | --- | --- | --- | --- | --- | --- | --- | --- | --- |
| Review identification | Reference | Publication Year | Journal | Impact Factor JCR | Time frame assessed | Databases | Number of included studies | Study design of included studies | Meta-analysis performed | Patients: total (n) |  |
| Abhari et al | 40 | 2019 | Healthcare Informatics Research | NA | 2012 to 2017 | MEDLINE, Web of Science, and Embase | 31 | Cohort studies, clinical trials, and meta-analysis reports | N | NA |  |
| Albahri et al | 37 | 2020 | Journal of Medical Sciences | 3.058 | 2010 to 2020 | Science Direct, IEEE Xplore, Web of Science, MEDLINE, and Scopus | 8 | Case control studies | N | NA |  |
| Alonso et al | 45 | 2018 | Journal of Medical Sciences | 3.058 | 2008 to 2018 | Google Scholar, IEEE Xplore, MEDLINE, Science Direct, Scopus, and Web of Science | 35 | NA | N | NA |  |
| Arani et al | 41 | 2018 | Acta Informatica Medica | NA | 2000 to 2018 | MEDLINE Embase, Cochrane Library, Scopus and Google Scholar | 30 | NA | N | NA |  |
| Bernert et al | 19 | 2020 | International Journal of Environmental Research and Public Health | 2.468 | Inception to 2018 | MEDLINE, PsychInfo, Web of Science, Embase and, Google Scholar | 87 | Non-randomized controlled studies, prospective and retrospective cohort studies, case series, cross studies | Y | 5,986,238 |  |
| Bridge et al | 17 | 2020 | American Journal of Respiratory and Critical Care Medicine | 17.452 | 1809 to 2017 | MEDLIN, CINAHL Plus, Cochrane Library, Web of Science, and ClinicalTrials.gov | 10 (9 Meta-analysis) | Prospective and retrospective cohort studies, double blind trial retrospective, | Y | NA |  |
| Burke et al | 25 | 2019 | Journal of Affective Disorders | 3.892 | 2018 | PsycINFO, PsycARTICLES, ERIC, CINAHL, and MEDLINE | 35 | Cross-sectional and cohort studies | N | NA |  |
| Chaki et al | 32 | 2020 | Journal of King Saud University Science | 3.819 | 2015 to 2020 | Scopus and MEDLINE | 107 | Case control Studies | N | NA |  |
| Davidson et al | 30 | 2020 | Journal of Pharmacokinetics and Pharmacodynamics | 2.461 | 1990 to 2019 | Embase, MEDLINE, and Scopus | 31 | NA | N | NA |  |
| El Idrissi et al | 18 | 2019 | International Journal of Information Management | 8.21 | 2000 to 2017 | IEEE Xplore, ACM Digital Library, MEDLINE and Science Direct | 38 | NA | N | NA |  |
| Fleuren et al | 26 | 2020 | Intensive Care Medicine | 17.679 | Inception to 2019 | MEDLINE, Embase.com, and Scopus | 28 | Cohort studies | Y | NA |  |
| Freeman et al | 11 | 2018 | Disaster Medicine and Public Health Preparedness | 0.977 | Inception to 2017 | MEDLINE, Embase, ISI Web of Science, BIOSIS, LILACS, CINAHL, Scopus, and NIH Reporter | 113 | NA | N | NA |  |
| Galetsia et al | 39 | 2019 | Social Science & Medicine | 3.616 | 2000 to 2016 | Web of Science and Scopus | 804 | NA | N | NA |  |
| Gonçalves et al | 36 | 2020 | BMJ Open Gastroenterology | NA | NA | MEDLINE, IEEE, ACM Digital Library, and Digital Bibliography Library Project | 33 | NA | N | NA |  |
| Harris et al | 27 | 2019 | PLoS One | 2.74 | 2005 to 2019 | MEDLINE, Embase, and Scopus | 53 40 were classified as Development studies and 13 were classified as Clinical | Prospective and retrospective cohort studies | N | NA |  |
| Layeghian et al | 42 | 2018 | Journal of Biomedical Informatics | 3.526 | 2000 to 2018 | MEDLINE, Science Direct, Google Scholar and SpringerLink | 75 | Case control studies, cases series and case reports | N | NA |  |
| Kannan et al | 35 | 2020 | Archives of Clinical Infectious Diseases | NA | 2013 to 2020 | MEDLINE, Scopus, Web of Science, Science Direct, and Google Scholar | 30 | NA | N | NA |  |
| Kavakiotis et al | 38 | 2017 | Computational and Structural Biotechnology Journal | 6.018 | 2011 to 2016 | MEDLINE and the DBLP Computer Science Bibliography | 103 | NA | N | NA |  |
| Klarenbeek et al | 24 | 2020 | Cancers | 6.126 | 2000 to 2019 | MEDLINE, Embase, Cochrane Library, IEEE, ACM Digital Library, and Web of Science | 9 | Cohort studies | N | 7985 |  |
| Kruse et al | 23 | 2016 | JMIR Medical Informatics | 2.577 | 2010 to 2016 | MEDLINE, CINAHL, and Google Scholar | NA | NA | N | NA |  |
| Li et al | 31 | 2019 | Diagnostics | 3.11 | 2009 to 2019 | Embase, MEDLINE, Cochrane Library, IEEE, Scopus, and Web of Science | 26 | Case control studies | N | NA |  |
| Librenza-Garcia et al | 12 | 2017 | Neuroscience & Biobehavioral Reviews | 8.33 | 1960 to 2017 | MEDLINE, Embase, and Web of Science | 51 | Cohort studies and Meta-Analysis | N | 17926 |  |
| Luo et al | 34 | 2015 | BMC Medical Informatics and Decision Making | 2.317 | Inception to 2015 | MEDLINE, Embase, CINAHL, Scopus, Cochrane Library, ACM Digital Library, IEEE Xplore, and OpenGrey | 32 | Case control, cohort and cross-sectional studies | N | NA |  |
| Murray et al | 44 | 2019 | Journal of Neuro Interventional Surgery | 4.46 | 2014 to 2019 | MEDLINE and Embase | 20 | Cohort studies, meta-analysis, and one randomized clinical trial | N | NA |  |
| Nielsen et al | 15 | 2018 | Ophthalmology Retina | NA | 2016 to 2018 | MEDLINE and Embase | 11 | NA | N | NA |  |
| Patil et al | 28 | 2019 | Journal of Oral Pathology & Medicine | 2.495 | 1990 to 2018 | MEDLINE, Embase, Scopus, Web of Science, and gray literature (Google Scholar, ProQuest, and OpenGrey) | 7 | Cohort studies, one case‐control study and one randomized clinical trial | N | NA |  |
| Pehrson et al | 29 | 2019 | Diagnostics | 3.11 | 2014 to 2018 | MEDLINE, Web of Science, Scopus, IEEE, and ACM Digital Library | 41 | NA | N | NA |  |
| Scardoni et al | 33 | 2020 | Journal of Infection and Public Health | 2.447 | Inception to 2018 | MEDLINE, and Embase | 27 | Prospective and retrospective cohort studies | N | NA |  |
| Shatte et al | 14 | 2019 | Psychological Medicine | 5.813 | 2004 to 2018 | PsycINFO, Cochrane Library, MEDLINE, IEEE Xplore, ACM Digital Library, Springer, Scopus, and Science Direct | 300 | Cohort studies, one randomized clinical trials and meta-analysis | N | NA |  |
| Sprockel et al | 13 | 2018 | Archivos de cardiología de México | NA | Inception to 2014 | MEDLINE, Embase, Scopus, IEEE/IET Electronic Library, ISI Web Of Science, Latindex, and LILACS | 35 | NA | N | NA |  |
| Tomaselli Muensterman et al | 16 | 2018 | Pharmacotherapy | 3.473 | 1879 to 2017 | MEDLINE, Cochrane database, Embase, and Ovid | 10 | Retrospective and prospective cohort studies | N | NA |  |
| Tripoliti et al | 21 | 2017 | Computational and Structural Biotechnology Journal | 6.018 | Inception to 2000 | MEDLINE, Scopus, Science Direct, Google Scholar, and Web of Science | 50 | Cohort studies | N | NA |  |
| Wang et al | 43 | 2020 | PLoS One | 2.74 | 1990 to 2019 | MEDLINE, and Web of Science | 18 | Cohort studies and randomized clinical trial | N | NA |  |
| Woldaregay et al | 20 | 2019 | AI in Medicine | 4.383 | 2000 to 2018 | Google Scholar, IEEE Xplore, DBLP Computer Science Bibliography, Science Direct, MEDLINE, Journal of Diabetes Science and Technology, and Diabetes Technology & Therapeutics | 55 | NA | N | NA |  |
| Yin et al | 22 | 2019 | Journal of the American Medical Informatics Association | 4.112 | 2010 to 2018 | MEDLINE, Web of Science, IEEE Library, ACM library, AAAI library, and the ACL anthology | 103 | Cohort and case control studies | N | NA |  |

| Review identification | Reference | Accuracy | AUROC | Precision | Recall | F-measure |
| --- | --- | --- | --- | --- | --- | --- |
| Abhari et al | 40 | x | X | X | x |  |
| Albahri et al | 37 | X |  |  |  |  |
| Alonso et al | 45 | X |  | X | X | X |
| Arani et al | 41 | X | X | X | x |  |
| Bernert et al | 19 | X | X |  |  |  |
| Bonnett et al | 17 | X |  |  | x | X |
| Burke et al | 25 | X |  |  |  |  |
| Chaki et al | 32 | X | X |  |  |  |
| Davidson et al | 30 |  | X |  | x |  |
| El Idrissi et al | 18 | X |  |  |  |  |
| Fleuren et al | 26 | X | X |  |  |  |
| Freeman et al | 11 |  |  |  |  |  |
| Galetsia et al | 39 |  |  |  |  |  |
| Gonçalves et al | 36 | X | X |  |  |  |
| Harris et al | 27 | X | X |  | x |  |
| Layeghian et al | 42 |  |  |  |  |  |
| Kannan et al | 35 |  |  |  |  |  |
| Kavakiotis et al | 38 | X | X |  |  |  |
| Klarenbeek et al | 24 |  |  |  |  |  |
| Kruse et al | 23 |  |  |  |  |  |
| Li et al | 31 | X | X | x | X |  |
| Librenza-Garcia et al | 12 |  | X | X | X |  |
| Luo et al | 34 | X | X | x | x |  |
| Murrax et al | 44 | X | X | x | x |  |
| Nielsen et al | 15 | X | X |  | x |  |
| Patil et al | 28 | X | X |  |  |  |
| Pehrson et al | 29 | X | X |  | x |  |
| Scardoni et al | 33 | X | X | X | X | X |
| Shatte et al | 14 | X |  |  |  |  |
| Sprockel et al | 13 |  | x | x | x |  |
| Tomaselli Muensterman et al | 16 | X | X |  |  |  |
| Tripoliti et al | 21 | X | X | X | X | X |
| Wang et al | 43 | X | X |  | x |  |
| Woldaregay et al | 20 | X | X |  |  |  |
| Yin et al | 22 |  |  |  |  |  |
