# Supplementary material for: Impact of Big Data Analytics on People’s Health: Overview of Systematic Reviews and Recommendations for Future Studies
Source: J Med Internet Res. 2021 Apr 13;23(4):e27275. doi: 10.2196/27275 (PMC8080139; doi:10.2196/27275)
Supplement: Multimedia Appendix 4 [file jmir_v23i4e27275_app4.docx]

| Multimedia Appendix 4 – Results and limitations of included systematic reviews | | | | | | |
| --- | --- | --- | --- | --- | --- | --- |
| **Review identification** | **Condition** | **Purpose of the study** | **Type of analytical tools used** | **Main results** | **Limitations** |  |
| Abhari et al (2019) | Diabetes Mellitus | To review AI algorithms and techniques for type 2 diabetes mellitus care with a specific focus on ML methods | Supervised ML) techniques (SVM, NB, RF, DT, ANN) | 1. 71% of papers applied ML (mostly SVM 21% and NB approaches 19%), 23% used multiple methods, and just 6% of them used the KB method 2. ML methods often achieve high accuracy due to looser assumptions regarding data distribution in comparison to other methods 3. The main clinical variables in running and designing ML models and systems for type 2 diabetes care, were body mass index, fasting blood sugar, blood pressure (systolic and diastolic), HbA1c, triglycerides, low-density lipoprotein, high-density lipoprotein, family history, and demographic variables 4. Models based on ML algorithms in type 2 diabetes mellitus care have been mainly focused on pre-diabetes screening and diagnostic outcomes, risk factor analysis, treatment, and complication categorization. 5. ML algorithms have been mainly used to classify diabetic prone cases for pre-diabetes, diabetes, and advanced diabetes based on the patients’ HbA1c level. 6. AI has often shown success in the prediction of related issues in type 2 diabetes mellitus. | 1. The focus of this review was clinical databases; therefore, more technical databases, such as IEEE and Scopus should be considered for further reviews. 2. Future investigation should focus on the effect of AI on clinical outcomes and its impact. |  |
| Albahri et al (2020) | Coronavirus Family | To obtain an overview of this critical virus, address the limitations of utilizing DM and ML algorithms, and provide the health sector with the benefits of this technique. | Supervised ML, topic modeling (LDA), case-based reasoning | 1. Decision tree, NB, and SVM algorithms were the most frequently used. 2. *k*-NN classifier is the best model for the two-class problems, and the decision tree and NB are the best models for multiclass problems. 3. The experimental results indicated that age and symptoms are the two dominant features for the prediction model and that healthcare staff are likely to survive. 4. The lack of studies on the recent outbreak of COVID-19 indicates the need and opportunity to apply AI for predicting such outbreaks. | 1. The lack of a large dataset in the academic literature for MERS-CoV is considered a challenging task because it hinders the understanding of viral patterns and features. 2. Other challenges are correlated with people and government responses to MERS-CoV that requires more new monitoring approaches and additional efforts compared with the traditional approach for controlling epidemic diseases 3. Another challenge with MERS-CoV is the large variation in symptoms that are mostly similar to common cold symptoms, with many other variations of diseases that may occur in cases but not in others. 4. Some patients have unique symptoms, and others have no symptoms at all. 5. Activists have generated huge and complex volumes of data that render its analysis impractical and difficult to predict using linear classifiers 6. The protection of citizens by the government and health agencies is a significant challenge because no specific vaccine exists for this virus to date and requesting people to undergo medical check-ups is difficult |  |
| Alonso et al | Mental Health | To present a review of the existing research works in the literature, referring to the techniques and algorithms of DM in mental health, specifically in the most prevalent diseases such as: Dementia, Alzheimer, Schizophrenia and Depression. | Supervised ML (including genetic algorithms), unsupervised clustering, association rules (a priori) | 1. 72 articles have been identified as relevant works of which 32% are Alzheimer’s, 22% dementia, 24% depression, 14% schizophrenia and 8% bipolar disorders. 2. Many of the papers show the prediction of risk factors in these diseases by the use of DM techniques. 3. The use of DM techniques applied to diseases such as dementia, schizophrenia, depression might be useful to the clinical decision, diagnosis prediction and improve the patient’s quality of life. 4. ML applications with neural networks, mini mental state examination and functional activities questionnaire methods have become useful to classify states of dementia and improve accuracy. 5. SVM approaches and NB are the most commonly used approaches applied for depression, but no performance results are given or discussed by review authors. | 1. A challenge when developing predictive clinical tools is to establish what information should be used. Genetic and brain imaging measures are possible sources of information and have generated interest. However, even if effective, the cost and time of data collection and processing may not be practical. |  |
| Arani et al (2018) | Multiple Sclerosis | To analyze applied reasoning methods and techniques in intelligent computer systems to help multiple sclerosis diagnosis | Supervised ML, unsupervised clustering, fuzzy logics, statistical analysis | 1. The rule-based method is one of the best applications of disease diagnosis compared to SVM, NB approaches. This method manages well-defined problems with knowledge-based texts. 2. By the use of natural language processing, medical records of patients are used in intelligent systems, providing the identification of multiple sclerosis patients in the primary steps of the clinical course of disease using simple algorithms. Moreover, this method increases diagnosis accuracy and the positive prediction value. 3. Fuzzy logic allows the software variables of the decision support system to be members of different sets, simultaneously and with different degrees. 4. The average sensitivity, specificity, and accuracy of reasoning methods were 91%,77%, and 86% respectively | 1. The number of selected articles in this review article was limited due to lack of availability of the full texts. 2. The calculated efficiency scales were different in various articles. 3. Evaluation results of system efficiency have been described qualitatively with phrases such as “increasing efficiency” and “accuracy is very good”. Therefore, the efficiencies of some applied methods and algorithms were not comparable with each other. |  |
| Bernert et al (2020) | Suicide | To identify and summarize original reports employing use of an AI and ML framework to predict suicidal behaviors as an outcome of risk | Supervised and unsupervised ML techniques (including self-organizing maps (SOM), principal component analysis (PCA) | 1. The majority of studies used supervised learning techniques (including ensemble learning methods, SVMs, NB approaches). 2. Results suggest high levels of risk classification accuracy (>90%) and area under the curve in the prediction of suicidal behaviors. 3. Natural language processing has been associated with suicidal behaviors, including studies considering acoustic features of speech to recognize risk within emergency department settings, text-based applications, or social media evaluation. 4. Among classification studies, the mean area under the curve was 0.814, while among those studies reporting accuracy values reached mean performance of 81.3%. | 1. Methods varied widely across reports, both with respect to ML methods and study quality 2. Incomplete reporting of test statistics (e.g., accuracy, AUC, sensitivity, specificity) and different methods for assessing and defining risk within diverse ML methods highlights need for improved reporting standards and a priori-designed studies. 3. Key parameters, such as PPV, AUPRC, and lead-time of the prediction which allows for decision-making about when to potentially act and intervene were also underreported 4. Challenges inherent in retrospectively analyzing health data for administrative and clinical purposes should also be noted, given the high number of studies using EMR. |  |
| Bonnett et al (2020) | Asthma | To identify studies which have developed such models, determine whether consistent and appropriate methodology was used and whether statistically reliable prognostic models exist. | Probabilistic approaches (NB), logistic regression, survival analysis, action points, and Classification and Regression Tree Analysis (CART). | 1. Five distinct types of model development methodology to predict future asthma exacerbation crises were identified (probabilistic approaches, logistic regression, survival analysis, action points, and classification and regression tree analysis) 2. Using ML approaches, the SVM had a slightly higher performance than compared to NB approach (sensitivity 84% and specificity 80% versus sensitivity 80%, specificity 77%, respectively). 3. Based on meta-analysis results, the diagnostic ability across all the models considered was 0.77 (95% confidence interval 0.73 to 0.80), showing that these models are some- what effective at predicting the risk of asthma exacerbations. | 1. The meta-analysis of heterogeneous studies was undertaken to investigate and demonstrate broad points and therefore the exact c statistic estimates are not of direct clinical relevance given the degree of study heterogeneity. 2. In particular, the summary statistic from the meta-analysis is to give an indication of the performance, rather than to be an accurate pooled estimate. 3. An additional limitation is the potential for the reviewer bias given that only a random subset of studies was reviewed by a second author. 4. Full text articles were cross validated, again without disagreements. |  |
| Burke et al (2019) | Mental Health | To review the existing literature on the application of ML techniques to predict self-injurious thoughts and behaviors | Supervised ML techniques | 1. Included articles were reviewed by outcome: suicide death, suicide attempt, suicide plan, suicidal ideation, suicide risk, and non-suicidal self-injury. 2. A greater prediction accuracy of self-injurious thoughts and behaviors was observed than in previous studies using traditional statistical methods. 3. Studies using ML for variable selection purposes have both replicated findings of well-known self-injurious thoughts and behaviors risk factors and identified novel variables that may augment model performance. 4. Among service members with baseline psychiatric hospitalization, ML model area under curve ranged from 0.71-0.89 in predicting suicide death. 5. ML models based on electronic medical records indicators to predict SA over varying timeframes (area under the curve = 0.80 to 0.84), with model performance increasing closer to time of suicide attempt (from 720 days to 7 days prior to the suicide attempt) 6. The performance of ML methods for suicide attempt varied substantially, with accuracy ranging from 67% to 94%, sensitivity ranging from 12% to 100% and specificity ranging from 39% to 99%., while performance for prediction of suicide ideation had accuracy from 80% to 91%, sensitivity from 63% to 88% and specificity from 62% to 94%. | 1. Relatively low paper sample size, inconsistent reporting procedures resulting in an inability to compare model accuracy across studies 2. Lack of model validation on external samples. |  |
| Chaki et al (2020) | Diabetes Mellitus | To perform an analysis of the detection, diagnosis, and self-management techniques of diabetes mellitus from six different facets (datasets of diabetes, pre-processing methods, feature extraction methods, ML-based identification, classification, and diagnosis of diabetes mellitus, AI-based intelligent diabetes mellitus assistant and performance measures) | Supervised Classifiers (ANN, SVM, NB, DT, LDA, RF, Ensembles, Fuzzy based methods), unsupervised clustering, and AI-Based agents | 1. Text-based, texture-based, fusion of shape-based, and color-based features are the most biased features of diabetes mellitus recognition algorithms and can yield promising outcomes for scientists operating in the area of ML based diabetes mellitus detection and diagnosis and classification. 2. Deep neural network and SVM reported better classification outcomes followed by random forest and ensemble classifier. 3. Convolutional neural network is mainly used in DL to automatically retrieve and identify diabetes mellitus data. | 1. The most critical challenge in ML-based methods is to find valuable features that can be given as inputs to ML algorithms to generate a classification architecture. |  |
| Davidson et al (2020) | Women Health | To assess the role of AI in women’s health, discover gaps, and discuss the future of AI in maternal health | Supervised ML Techniques | 1. There are three areas where AI methods could be used to improve our understanding of pharmacological effects of pregnancy, including: (a) obtaining sound and reliable data from clinical records; (b) designing optimized animal experiments to validate specific hypotheses and (c) implementing decision support systems that inform decision-making. 2. The largest literature gap is with regards to using AI methods to optimize translational studies between animals and humans for pregnancy-related drug exposures | 1. Most studies included cross-validation, but external validation was limited. This impedes on generalizability of the studies. 2. Few applications of AI are focused on pharmacological treatment. 3. This review does not assess the role of AI methods in lactation studies. |  |
| El Idrissi et al (2019) | Diabetes Mellitus | To identify, analyze and synthesize the studies dealing with the use of DM predictive techniques in Diabetes self-management | Supervised ML  techniques  (including fully adaptive regularized learning (FARL) and ridge regression (RR) | 1. Prediction of blood glucose levels was the most common clinical task regarding diabetes self-management, followed by regulation and control of blood glucose levels, detection of hypoglycemic episodes, and estimation of insulin dosage. 2. ANNs were the most frequently used predictive technique which along with autoregressive models, yield highest accuracy rates. 3. SVMs, Kalman filter and subspace were also frequently used among created models, but mostly without detail of performance values. | 1. Had some threats to validity (search strategy related selection bias, data extraction bias, publication bias), however taken steps to mitigate them. |  |
| Fleuren et al (2020) | Sepsis | To systematically review the use of ML to predict sepsis in the intensive care unit, hospital wards, and emergency department | Supervised  ML techniques (including DL) | 1. The majority of papers were developed in the intensive care unit, followed by hospital wards and emergency department. 2. For the prediction of sepsis, diagnostic test accuracy assessed by the AUROC ranged from 0.68 to 0.99 in the ICU, to 0.96 to 0.98 in-hospital wards and 0.87 to 0.97 in the emergency department. 3. Varying sepsis definitions limit pooling of the performance across studies. 4. In the multivariate analysis, temperature, lab values, and model type contributed most to model performance. | 1. AUROC was pragmatically chosen as a summary measure, while it may underperform in the setting of imbalanced datasets. Nonetheless, it was the summary measure most frequently reported; other measures would have eroded the possibility to compare performance across studies. 2. No contingency tables were feasible for the majority of papers as the necessary data were too infrequently reported and very few papers reported measures of uncertainty such as confidence intervals or standard deviations. |  |
| Freeman et al | Disasters | To describe the current state of the art in information and communication technology and big data applications in disaster settings and to determine potential avenues for future research and application | Geographic information systems, social media tools – Other ML Algorithms not specified | 1. Nearly 80% of studies assessing the application of big data in disaster management covered only the response phase of disasters and only 15% of the studies addressed disasters in low- and middle-income countries. 2. The 4 most frequently mentioned information and communication tools were geographic information systems, social media, patient information, and disaster modeling. 3. Most studies (64.4%) listed either clinical first responders or community members as the main intended users of big data tools during disaster. 4. With regards to the use of big data tools by disaster type, most studies were related to hurricanes and earthquakes (38.9%). | 1. Given the complex relationship of causes contributing to complex humanitarian emergencies, it is possible that our search strategy did not capture studies of tools used in complex humanitarian emergencies that were cataloged under terms associated with warfare or conflict rather than disasters. 2. This research addressed peer-reviewed scientific literature, while the grey literature may provide examples of novel ICT or big data tools that have not been the subject of rigorous research. 3. This study addressed the scope of ICT and big data use and not the quality of the studies themselves and did not assess the quality of the included studies. 4. There may be studies in non-English languages that have not been reviewed in the major English-language databases used in our research |  |
| Galetsia et al | Challenges, values and opportunities of big data analytics | To systematically review scholarly publications related to big data analytics in health in order to identify the organizational and social values along with associated challenges | Not specified | 1. 10 main values associated with the use of big data analytics in patient care were highlighted: 1. New approaches to diagnosis for personalized healthcare, 2. Replacing/supporting human decision-making with automated algorithms, 3. Innovating new business models, products, and services, 4. Enabling experimentation to discover needs, expose variability, and improve performance, 5. Coordination of healthcare information, 6. Creating efficiency, 7. Identify patient care risk, 8. Offering customized actions by segmenting populations, 9. Achieving cost-effectiveness and 10. Protecting privacy 2. The majority of researchers expect health organizations and patients to gain value from the “personalized innovative medical approaches (35.6%) | 1. For the systematic review, articles were only obtained from Web of Science® and Scopus. 2. This study uses as a methodology a systematic literature review approach which could be broadened to include many other aspects of health sociology, such as the effects of health data commercialization, the changing environment of labor etc. 3. Sociology can shed light on some of the identified challenges such as data ownership, revealing where points of exploitation occur, and on issues of healthcare providers' responsibilities and their capacity to enforce or discourage certain behaviors. |  |
| Gonçalves e Gonçalves et al (2020) | Gastric Tissue Diseases | To map, inspect and discuss the literature content regarding the applications of DL methods based on gastric tissue medical images. | Deep Leaning (CNN mostly, but also Recurrent, Residual and neural networks) | 1. The majority of papers were developed in the intensive care unit, followed by hospital wards, and the emergency department 2. 15 articles related to histology, 11 were related to the application of DL to endoscopy images, immunohistochemistry or pathology images 3. 5 publications applied DL methods to stomach X-ray images. 4. There are few studies with direct application in gastric cancer, either for classification, detection or segmentation. 5. DL models were built under training with histological or endoscopic images. 6. CNN is a widely used and state-of-the-art model. 76% of articles proposed applications of CNN for the diagnosis of gastric cancer or the detection of lesions in gastric tissue. 7. Other approaches used were fully connected networks, with two applications: segmentation of gastric tumors and gastric cancer. 8. Recurrent neural networks with one application: predicting survival rate after gastric surgery; deep residual networks with three applications: prediction of microsatellite instability in the gastrointestinal cancer, gastric cancer segmentation, and classification of gastric cancer type (intestinal and diffuse type); and generative adversarial networks with one application: generation of synthetic and anonymous images of gastritis. 9. Prediction of sepsis, diagnostic test accuracy assessed by the AUROC ranged from 0.68–0.99 in the ICU, to 0.96–0.98 in-hospital and 0.87 to 0.97 in the ED. 10. In the multivariate analysis, temperature, lab values, and model type contributed most to model performance. | Not available |  |
| Harris et al (2019) | Pulmonary Tuberculosis | To evaluate the evidence base with regards to the estimation of the diagnostic accuracy of computer-aided detection, including assessing potential for bias, and if appropriate, to calculate pooled estimates of area under the receiver operating characteristic curves, sensitivity, and specificity. Secondary objectives were to evaluate study-level factors associated with diagnostic accuracy; including those related to the design of the study, and the type of software used | Supervised ML techniques including deep learning (CNN) | 1. 17% of studies employed DL methods while the remaining 83% used ML approaches. 2. 83% of the development studies reported measures of accuracy for index tests. 3. Of the 33 references that did include accuracy assessments, the AUC ranged from 0.78 to 0.99, sensitivity from 0.56 to 0.97, and specificity from 0.36 to 0.95. 4. For the triage studies that used a microbiologic reference, the sensitivity ranged from 0.86 to 1.00, and specificity ranged from 0.23 to 0.69. 5. In the screening studies, sensitivity ranged from 0.53 to 0.89 and the specificity ranged from 0.56 to 0.98. 6. While not statistically significant, we found that the median AUC of studies using a human reader as the reference standard were higher than those studies using a microbiologic reference standard of 0.88 [0.81–0.90] versus 0.77 [0.67–0.89] respectively (p = 0.16). | 1. Meta-analysis was not completed due to the methodological heterogeneity, the lack of standardized threshold scores, and the variability of software versions used. 2. While the software achieved high sensitivities (0.85 to 1.0), there was a large degree of variability in the reported specificities (0.23–0.69). 3. The analysis in some studies was performed on CXRs from datasets or sites that may have also contributed to training the software, potentially resulting in an overestimation of the predictive power. 4. Because the populations studied had very high HIV and TB prevalence, the results may have limited generalizability to other populations |  |
| Javan et al (2018) | Cardiac arrest | To evaluate the capability and performance of ML techniques in predicting the risk of cardiac arrest and to offer an integrative framework to synthesize the researches in this field. | Supervised ML techniques including Deep learning and ensembles. Supervised an Unsupervised Feature Selection has been employed | 1. A considerable number of included papers (52/75) used the para-clinical findings of the heart which included the parameters of ECG, heart rate variability, echocardiography, cardiopulmonary exercise testing. 2. The most frequently used parameters were related to ECG and HRV signals. 3. Evidence suggests that changes in vital signs occur a few hours before the cardiac arrest happens. 4. The other types of used variables were demographics (24/75), laboratory test results (24/75), history of illness or treatments (18/75), medications (9/75), hospitalization history (5/ 75) and existing risk scores 5. In studies related to cardiac arrest prediction, supervised ML techniques were used for feature selection, classification, prediction or evaluating the efficiency of some parameters. 6. Feature selection, regression techniques, SVMs, instance-based techniques, neural networks, decision trees, ensemble- classifiers, association rule mining, reinforcement learning techniques, fuzzy methods, Bayesian and hybrid algorithms. 7. The highest values of 98%, 100%, 100% and 100% were obtained for AUROC, sensitivity, specificity and PPV using a SVM algorithm among the papers with the aim of classifying normal and cardiac arrest. 8. ML techniques used for classification produced the highest overall performance. 9. KNN algorithm provides a better overall performance (greater than 90% in 75% of cases). 10. The logistic regression algorithm appears to have generated poorer overall results compared to the rest of the algorithms. | 1. The models are developed and evaluated with limited hospital data; therefore, the generalizability of the models to other patient populations and health systems is unknown. 2. Despite the improvements, the rate of false alarms is still high. 3. None of the studies considered lifestyle and social factors in CA prediction. 4. Most studies did not mention a specific reason for choosing an ML technique. 5. Since studies are not designed as RCT, it is unclear whether the proposed automatic systems are usable in real clinical settings and can improve the patient's survival rate. |  |
| Kannan et al (2020) | SARS-CoV-2 | To highlight the importance of AI and ML techniques that can speed up the path to the discovery of a possible cure for COVID-19 as well as to assess the interactions between viromics and AI, which can hopefully find a solution to this pandemic | Deep Leaning (CNN, RNN and Truncated Back Propagation Through Time) | 1. There are 4 main applications of AI and ML for COVID-19: a. AI to Spot Patterns and Changes; b. Viral Host Prediction with DL, c. Detecting COVID-19 in X-Ray Images with AI Tools and d. Exploring SARS-CoV-2 Spike Glycoprotein with Python Tools. 2. DL-based drug screening for novel coronavirus is also a reality to predict the interactions between proteins and ligands, helping to verify which drug combinations work preferably well in response to the virus 3. AI scientists in Wuhan developed this method to identify the intensity of infections with factors such as age, gender, etc. | Not available |  |
| Kavakiotis et al (2017) | Diabetes Mellitus | To conduct a systematic review of the applications of ML, DM techniques and tools in the field of diabetes research with respect to a) Prediction and Diagnosis, b) Diabetic Complications, c) Genetic Background and Environment, and e) Health Care and Management with the first category appearing to be the most popular | Supervised  ML Classifiers | 1. Chronic diabetic complications include heart failure, diabetic neuropathy, nephropathy, retinopathy, and diabetic foot. 2. Moreover, both insulin resistance and hyperglycemia have been implicated in the pathogenesis of diabetic dyslipidemia 3. It is worth noting that diabetes complications are far less common and severe in people with well-controlled blood glucose levels. 4. Many of those complications have been studied through ML and DM applications. 5. DM techniques can help a) recommend and improve effective medication, b) predict and suggest more personalized medications, c) design more effective blood glucose lowering factors, d) improve insulin planning and dosage, and e) implement drug administration in a more specific manner. | Not available |  |
| Klarenbeek et al (2020) | Oncology | To explore the impact of higher level computerized clinical decision support systems on quality of care in oncology, operationalized in terms of process outcomes, guideline adherence and clinical outcomes | Computerized clinical decision support systems (CDSS) | 1. The included studies demonstrated significant improvements of higher level computerized clinical decision support systems on process outcomes and guideline adherence across diverse settings in oncology. 2. Implementation of a computerized clinical decision support systems was shown to positively impact the following process outcomes: a. Physician-prescribing behavior: studies showed that initial decisions in breast cancer management were modified in 31% of cases after implementation of a CDSS. Whatever the motivation for change, it was always directed towards an improvement in patient management. b. Frequency of pain assessment: studies showed that frequency of nursing pain assessments within 24 hours after admission was significantly higher in the post-implementation group compared to the pre-implementation group (12.0 vs. 7.4 p<0.001). Increased frequency of nursing pain assessment is associated with improved pain outcomes. c. Healthcare costs: one study suggests that the use of computerized clinical decision support systems resulted in a significant reduction in total costs for stage IV non-small cell lung cancer patients one year after diagnosis, by approximately $17,000 (from $69,122 before to $52, 037). d. Clinician’s workload: one study calculated that on average a clinician needs 64 minutes per patient per year in the follow-up of colorectal cancer. Clinicians’ workload was significantly reduced to 23 minutes per patient per year after implementation of the computerized clinical decision support systems, saving more than 40 minutes per patient per year during follow-up. | 1. Higher level CDSS may be misclassified as low level during the screening process due to limited or unclear information. Subsequently, exclusion of misclassified CDSSs cannot be rule out. 2. General observations on higher level CDSSs are limited due to small numbers of studies included and heterogeneity in interventions, populations, settings and outcomes. 3. Publication bias and selective reporting cannot be excluded. 4. In the evaluation of CDSSs a double-blind design is not possible, as caregivers have to use the system. Therefore, even a well-designed RCT evaluating a CDSS will be rated low level of evidence. |  |
| Kruse et al | Challenges and Opportunities | To objectively review articles and studies published in academic journals in order to compile a list of challenges and opportunities faced by big data analytics in healthcare in the United States. Particular emphasis was paid to age-related applications of big data | Not specified | 1. The top challenges were issues of data structure, security, data standardization, storage and transfers, and managerial skills such as data governance. 2. The top opportunities revealed were quality improvement, population management and health, early detection of disease, data quality, structure, and accessibility, improved decision making, and cost reduction. 3. Nine themes emerged under the category of challenges: data structure, security, data standardization, data storage and transfers, managerial issues such as governance and ownership, lack of skill of data analysts, inaccuracies in data, regulatory compliance, and real-time analytics. 4. Fourteen themes emerged under the category of opportunities: improve quality of care, managing population health, early detection of diseases, data quality, structure, and accessibility, improve decision making, cost reduction, patient-centric care, enhanced personalized medicine, globalization, fraud detection, and health-threat detection. | Not available |  |
| Li et al (2019) | Pulmonary nodule | To investigate how DL performs for pulmonary nodule detection and/or classification of CT scans when the method is tested on datasets that are not from LIDC-IDRI. Furthermore, the study aim was to investigate whether the performance of DL is reduced when the algorithm is tested on a dataset that is different from the training dataset | Supervised Deep Learning | 1. Three different DL algorithms were mentioned in the studies: convolutional neural network, massive training ANN, and deep supervised denoising autoencoder architecture based on extreme learning machines. 2. Among studies providing classification performance results, studies reached a sensitivity between 75.6% to 85.6% and specificity between 80.1% to 98.7%. 3. For studies that only reported results on classification performance, five studies tested on local, independently obtained datasets. All studies provided reports of accuracy, which ranged between 68% to 92%. 4. Algorithms with convolutional neural network architecture reached accuracies between 68% to 99.6% on classification and 80.6% to 94% on detection. 5. No tendency of reduced performance was observed for the algorithms trained and tested on different datasets compared to the algorithms tested and trained on the same type of dataset. 6. Accuracy results for studies that tested and trained on same type of dataset were between 68% to 96.3%, while accuracy results from studies that tested and trained on different types of datasets were between 79.5% to 93.9% | 1. The heterogeneity of the included studies was a limitation of this review, this prevented performing a meta-analysis to statistically compare the performance of DL algorithms 2. There may be a risk of publication bias in the included studies |  |
| Librenza-Garcia et al (2017) | Bipolar Disease | To show ML techniques are likely to support important clinical decisions in the forthcoming years | Supervised ML (mostly SVM) | 1. Most studies used multiple levels of biological data to distinguish the diagnosis of bipolar disorder from other psychiatric issues (schizophrenia, unipolar depression, healthy controls and other conditions); 2. Several studies used structural neuroimaging (11), functional neuroimaging (13), genetic analysis (5), and encephalographic measures (4). 3. Some studies focused on predicting clinical outcomes (depression relapse, suicide, mood changes), while other studies focused on the prediction of treatment response or adverse effects. 4. ML can aid clinicians by improving risk assessment and by allowing early detection of those at risk for bipolar disorder. 5. Among classification studies using structural neuroimaging, accuracy values ranged from 54.76% to 100%, while studies using functional neuroimaging had accuracy between 52.13% to 92.07%, genetic analysis had accuracy between 61% to 73.4% and electroencephalographic studies had accuracy between 62.37% to 92.7%. 6. Feature selection seems to play an important role on the final accuracy of these models, as neuroimaging studies tend to have few subjects but can produce a large number of features. 7. Neuroimaging studies, besides helping us to better understand the pathophysiology of psychiatric disorders, may also help differentiate bipolar disorder from healthy controls and other psychiatric diagnosis. 8. ML techniques may also be used to assess individuals at risk, such as bipolar offspring, transforming data into applicable information about the individual risk of having a future diagnosis. 9. ML may allow us to develop personalized interventions to prevent the transition from prodromes to full-blown illness among high- risk patients. 10. Suicide, hospitalizations and episode relapse could also be predicted with reasonable accuracy by using ML models. 11. Emerging data also suggests that integration with mobile devices and social media could prove a useful resource to recognize a subject mood state, allowing the clinician to be advised prior to the onset of a mood episode in real time. | Not Available |  |
| Luo et al (2015) | Asthma | To review prediction diagnosis and prevention models of asthma | Supervised  ML (mostly Logistic regression), Clinical Indices | 1. With rare exceptions, existing predictive models for asthma development in children were developed using small data sets including (typically much) fewer than 2000 children. 2. Most models (17 of 23) for predicting asthma development for the general child population have low accuracy, typically with a sensitivity, positive predictive value, or area under curve much less than 80 %. 3. Most models (five of six) for predicting asthma development in children in the primary care setting have low accuracy, typically with an area under the curve much less than 80 %. 4. In general, a predictive model’s accuracy improves as the training data set becomes larger, particularly if the model uses many predictors. 5. By using an exhaustive set of variables coupled with a large number of children, we are likely to further improve the predictive models’ accuracy. 6. ML methods such as SVMs and random forests often achieve higher prediction accuracy than risk score, combination of risk factors, and logistic regression. 7. There are several potential approaches for improving accuracy, including ML methods, using large data sets and exhaustive variable sets, and focusing on a child population with a high prevalence of future asthma development. | 1. Excluding articles not written in English may have missed predictive models in other languages. 2. There may be other predictive models that have never been published. 3. Few studies directly compare predictive models on the same child population. (Performance metrics such as the AUC should not be used to directly compare predictive models across different child populations.) 4. There is no clear gold standard for the prediction target of asthma development in children. |  |
| Murray et al (2019) | Ischemic Stroke and large vessel occlusion | To review the current literature and new ML diagnostic technology for non-contrast CT, CT angiography, and CT perfusion images | Supervised  ML (mostly RF and CNN) | 1. AI use in acute large vessel occlusion stroke diagnostics and triage falls under three categories: automatic stroke core and penumbra size and mismatch quantification, detection of vascular thrombi or occlusions, and prediction of acute complications. 2. Reported sensitivity of AI algorithms range from 45% to 98%, mean 68%, and specificity ranges from 57% to 95%, mean 81%. 3. Use of a convolutional neural network for a combined asymmetric middle cerebral artery territory hypodensity and dense vessel detection may have higher performance; however, only area under the curve metrics is reported (receiver operating characteristic between 92% to 96%). 4. These large vessel occlusion detection studies variably report algorithm performance compared with individual humans, with broad sensitivities of 67% to 98% and area under the curve ranging from 85% to 93%. 5. AI algorithms are also used for acute prognosis prediction. This may facilitate immediate treatment planning | 1. Systematic and standardized methods for validation and comparison of this class of tools are needed. |  |
| Nielsen et al (2018) | Diabetes Mellitus | To gather the existing research on the use of DL and assess whether its application for automated diabetic retinopathy classification using fundus images could potentially be implemented in screening patients with diabetes | Supervised Deep learning (mostly CNN, including Hybrid models combining with CNN and RF) | 1. With regards to the use of DL methods in classifying full-scale diabetes retinopathy in retinal fundus images of diabetic patients, sensitivity values varied between 80.28% to 100%, while specificity varied between 84% to 99%. Accuracy ranged between 78.7% to 81%. 2. Incorporating DL methods could have many possible benefits to diabetes retinopathy screening programs around the world. 3. 78% of patients preferred an automated DL evaluation over human grading. 4. It may reduce the workload of clinicians, diminish intra- and inter-rater variability and provide a better cost-benefit balance. 5. The one-time purchase of a computer employing DL to diagnose diabetes retinopathy may be a more affordable and accessible eye care service to implement, compared to that of paying a doctor to continuously screen patients. 6. DL methods could be incorporated into a screening program in several ways (via semi-automated screening model or using a fully automated screening model). | 1. Target conditions vary, referable DR and DR of any severity 2. All studies have human graders, with different professions and would be expected to have different knowledge of image grading - risk of bias assessment. 3. In many of the included studies, parameters as age, sex, ethnicity, etc. were not described, as the datasets used were de-identified, this do not represent patients from a real-world. 4. The majority of the included studies did not report upgradable images, and among the few that did, only one accounted for them. |  |
| Patil et al (2019) | Head and Neck cancer | To evaluate the existing literature and assess the application of ML of genomic data in head and neck cancer | Supervised ML (mostly SVM, some ANN) | 1. Among the included studies, the accuracy rates for ML techniques ranged from 56.7% to 99.4%. 2. The majority of studies employed SVMs as ML techniques, while one study utilized multiple ML techniques. 3. Two studies reported that they used NanoString elements technology to assess the gene expression data 4. In addition to assessing genomic data, other variables were also analyzed including demographic data, clinicopathological data, and tumor status data. | 1. Smaller sample size was a common problem in almost all studies. It is important to have a sufficiently large dataset when classification schemes are used for modeling a disease. 2. A smaller training dataset leads to misclassifications and may result in unstable and biased models. |  |
| Pehrson et al (2019) | Lung nodules | To provide an overview of the literature available on ML algorithms applied to the Lung Image Database Consortium Image Collection (LIDC-IDRI) database as a tool for the optimization of detecting lung nodules in thoracic CT scans | Supervised  ML (mostly SVM) including Deep learning (mostly CNN) | 1. The majority of feature-based algorithms achieved an accuracy >90% compared to the DL algorithms that achieved an accuracy in the range of 82.2% to 97.6%. 2. The algorithms that applied a SVM classifier reached a range of accuracy of 68.4% to 99.0%, sensitivity of 55.0% to 98.6%, specificity of 87.5% to 98.2%, and an area under the curve of 0.905 to 0.998. 3. The convolutional neural network architecture reached an accuracy of 82.2% to 97.6%, sensitivity of 83.1% to 96.6%, specificity of 71.4% to 98.2%, and an area under the curve of 0.87% to 0.98%. 4. Deep convolutional neural networks reached an accuracy of 89.0% to 89.5% and a sensitivity of 84.2% to 87.1%. 5. In conclusion, ML and DL algorithms are able to detect lung nodules with a high level of accuracy, sensitivity, and specificity using ML, when applied to an annotated archive of CT scans of the lung. | 1. The general architecture of feature-based and DL algorithms differs. The specific architecture within the two groups also differs—the architectures and restrictions are set by the author |  |
| Scardoni et al (2020) | Infectious Diseases Control | To retrieve, quantitatively pool and critically appraise the available evidence on the development, implementation, performance and impact of ML-based healthcare-associated infection detection models | Supervised ML (mostly SVM, a few RF and ANN) | 1. There is moderate evidence that ML based models perform equal or better as compared to non-ML approaches and that they reach relatively high-performance standards. 2. 63% of ML approaches were classified as predictive and 37% as retrospective. 3. Overall, for ML based models for central line-associated bloodstream infections surveillance models tested in Parreco et al. had: (i) high specificity and negative predictive value, low sensitivity and positive predictive value, and had lower area under the receiving operator curve as compared to Beeler et al. 4. With regards to studies for sepsis surveillance, studies reported ML based models to detect sepsis more accurately than traditional clinical scores, retrospectively for epidemiological and performance evaluation purposes, and prospectively for preventive real-time evaluations. 5. Overall, ML models’ sensitivity ranged between 19% to 92%, specificity ranged between 72% to 96% and accuracy between 70% to 96% for healthcare associated infections. | Not available |  |
| Shatte et al (2019) | Mental Health | To synthesize the literature on ML and big data applications for mental health, highlighting current research and applications in practice | Supervised and Unsupervised ML Techniques (no dominant technique) | 1. Most commonly used ML techniques included supervised learning and classification approaches (such as SVMs, NB, decision trees), unsupervised and clustering approaches (such as k-NN, k-means clustering), text analysis, sentiment analysis and novel techniques, including techniques based on DL. 2. Mental health applications for ML techniques were identified in four key domains: (i) detection and diagnosis of mental health conditions; (ii) prognosis, treatment and support; (iii) public health; and (iv) research and clinical administration. 3. Predominantly, research has focused on the benefits of ML to improve detection and diagnosis of mental health conditions including depression, Alzheimer’s disease, and schizophrenia. 4. There has also been growing interest in the application of ML to other areas of mental health research, including the use of ML to improve administration and research methods, treatment and support of mental health conditions, studies of public health trends, and investigations into the behaviors of support communities online. 5. The majority of papers reviewed focus on diagnosis and detection, particularly on depression, suicide risk and cognitive decline. There is significant scope to explore whether ML can have similar accuracy in the detection and diagnosis of other mental health conditions, such as anxiety disorders, eating disorders, and neurodevelopmental disorders. | 1. Restrictions in the search methodology may have resulted in relevant articles being missed, e.g., broad search terms and the exclusion of non-peer reviewed literature |  |
| Sprockel et al (2018) | Acute Coronary Syndrome | To perform a systematic review of diagnostic test studies which evaluate the application of intelligent systems in the diagnosis of acute coronary artery disease, that might be useful in emergency wards | Supervised ML (mostly SVM, a few RFs and ANNs) | 1. 37% of studies used some intelligent system to perform the diagnosis of any acute coronary artery disease, while 62% were directly associated with only myocardial infarction. 2. Among included studies, 21 utilized clinical features and ECG results during hospital admission for performing its models, while one 2 studies used myocardial injury biomarkers. 3. Most intelligent systems used a set of patient’s information (clinical characteristics, ECG, myocardial biomarkers) along with any stratification score strategy. 4. In the vast majority of assessed models, high precision levels for diagnosis were observed, with greater performance being associated with neural networks (96% to 98% of accuracy). 5. By the use of different intelligent systems, accuracy ranged from 68.8% to 96.6%, sensitivity ranged from 52.4% to 100% and specificity ranged from 39% to 99%. | Not available |  |
| Tomaselli Muensterman et al (2018) | QT interval prolongation | To assess the effectiveness of predictive analytics for identification of patients at risk of drug induced QTc interval prolongation and to discuss the efficacy of incorporation of predictive analytics into clinical decision system tools in clinical practice | Clinical Decision Clinical Support Systems (CDSSs) | 1. Among included studies of predictive analytics tools to predict risk of or identify patients with QTc interval prolongation, sensitivity ranged from 67% to 99.8% and specificity ranged from 25.6% to 88%. 2. One group developed a risk score that includes information regarding genetic polymorphisms; this risk score significantly predicted the occurrence of Torsade’s de Pointes. 3. Clinical decision system tools have been developed to alert clinicians when patients are at moderate or high risk for developing QTc interval prolongation or to alert clinicians when patients have already developed QTc interval prolongation. | 1. Future research is required to determine the value of developing a risk score that combines genetic information with other known risk factors for QTc interval prolongation. |  |
| Tripoliti et al (2017) | Heart Failure | To present the state-of-the-art of the ML methodologies applied for the assessment of heart failure | Supervised ML Techniques (no predominant method) | 1. Most studies used heart rate variability in heart failure detection, with accuracy ranging between 83.65% to 96.39%, sensitivity ranging from 58.62% to 100% and specificity between 82% to 100%. 2. By the use of ML methods, the etiology or the subtypes of HF can be estimated using classification trees, bagged classification trees, random forests, boosted classification trees and SVM approaches. 3. The assessment of severity of heart failure patients has been associated with ML methods, in special by the use of SVM methods, fuzzy genetic, decision trees, random forest and remote telemedical monitoring, reaching varied accuracy (58.57% to 100%), sensitivity ranging from 73.30% to 100% and specificity ranging from 36.4% to 100%. 4. Studies reporting prediction of adverse events has been attempted over the last number of years, along with models predicting destabilizations, re-hospitalizations, and mortality. | 1. The utilization of different sources of data in each one of these studies limits their comparison, unlike then methods that detect HF, by utilizing HRV measures, that is applied to publicly available datasets commonly used in all studies. 2. All the studies addressed the issue as a two-class classification problem and did not take into consideration the patients belonging to the so called “gray zone” |  |
| Wang et al (2020) | Stroke | To identify gaps in the literature, critically appraise the reporting and methods of the algorithms and provide the foundation for a wider research program focused on developing novel ML based predictive algorithms in stroke care | Supervised ML Techniques (mainly and SVM, RF, DT, LR and ANN) | 1. All the included studies focused on developing new models using ML whilst no study validated existing ML based predictive models on independent data. 2. Mortality was the most frequently predicted clinical outcome. Studies focused on mortality at different time points during follow-up, including short term (10 days, 30 days, 2 months, 3 months, 100 days) and long term (1/3/5 years), and the most used models for mortality prediction were ANN, NB, SVM, decision trees and random forest. 3. There was no apparent relationship between the algorithms used and the sample size or number of features. 4. Six included studies reported that ML models outperformed the compared regression models and five studies concluded that there was no significant difference between the ML and statistical models. | 1. Studies not published in English and not in MEDLINE and Web of Science may be missed |  |
| Woldaregay et al (2019) | Diabetes Mellitus | To develop a compact guide regarding modeling options and strategies of ML applications and their hybrid system focusing on blood glucose prediction in type 1 diabetes mellitus | Supervised  ML techniques (mainly ANNs (simple Feed Forward NNs) and RNNs as well as SVM) | 1. Blood glucose dynamics among diabetic patients is influenced by various factors, such as the amount of insulin injection, the quantity of carbohydrate intake, the level and extent of physical activity, past readings of blood glucose, stress (emotional feelings), any type of illness, alcohol consumption, smoking, menstruation, and others. An ideal blood glucose predictor should incorporate all of these confounding variables for better estimation of the individual blood glucose levels. 2. For blood glucose prediction, feed-forward neural networks are the most used techniques (20%), followed by hybridization of the physiology-based models, recurrent neural networks (RNN) and SVMs approaches. 3. Physical activity has a significant effect on blood glucose dynamics. 4. Emotional stress or physiological stress caused by illness and infections could affect blood glucose homeostasis due to hormonal changes in the body. | 1. The lack of a well-defined approach to estimate carbohydrate intake, which is mainly done manually by the individual users and is prone to an error that can severely affect the predictive performance. 2. There is the lack of a universal approach to estimate and quantify the approximate effect of physical activities, stress, and infection incidence on the BG level. Almost all the studies have quite different approaches, and this poses a challenge in terms of regarding one approach as universal. 3. None of the researchers have assessed model predictive performance during stress and infection incidences in a free-living condition, which should be taken into account in future studies. |  |
| Yin et al (2019) | Several Conditions | To systematically review the effectiveness of applying ML methodologies to user-generated content for personal health investigations | Supervised  ML  (mainly LR, SVM, NB, Ensembles and some Deep Learning techniques) | 1. Some studies aimed to identify health problems, symptoms, and treatments, as well as classify users into treatment versus control by the use of ML. 2. Other studies focused on predicting the occurrence of a health issue, including learning posting patterns and their capability of predicting a health issue or event, along with studies evaluating posts about a health issue on social media and reports from authorities. 3. Relying on predefined semantic vocabularies, studies showed that users with mental health problems (eg, postpartum depression) in online environments (eg, Twitter, Facebook, Reddit) often expressed negative feelings and emotions, such as hopefulness and anxiety and exhibited lower social engagement and activity. 4. Social media analysis helped identify the characteristics of users with eating disorders or anorexia, including young age, high social anxiety, self-focused attention, deep negative emotions, and increased mental instability. 5. The datasets applied in these studies were mainly created through 3 methods: (1) snowball, (2) funnel, and (3) random sampling. 6. Popular off-the-shelf ML models were logistic regression, SVMs, NB, ensemble learning, and DL. 7. The most investigated problems were mental health and cancer. 8. Common health-related aspects extracted from user generated content were treatment experience, sentiments and emotions, coping strategies, and social support. | 1. Many ML-related keywords in the search queries were included to cover as many related publications as possible. However, this process might miss some studies that failed to mention such terms. 2. 345 workshop articles were removed before screening eligible publications and 2 after full article review. 3. Many studies that focused on public health and ADRs but neglected to investigate or discuss personal health were removed. 4. Some studies that investigated immunizations and performed opinion mining were excluded because there was no further investigation on their impact on personal health. 5. The group finding was cancer, eating disorder, sleep issues, mental health, vaccines, and others. however mental health and cancer were the 2 most popular studied health issues, and were investigated in 39 and 15 studies, respectively. |  |

CNN: convolutional neural network; k-NN; K-Nearest Neighbour, ML: machine learning, AI: artificial intelligence; DM: data mining; SVM: support vector machine; DL: deep learning; NB: naive Bayes; ANN: artificial neural network; BG: blood glucose; HF: heart failure; CT: computer tomography; MERS-CoV: Middle East respiratory syndrome; AUC: area under curve; AUPRC: area under precision recall curve; PPV: predictive positive value; ICU: intensive care unit; ED: emergency department; HIV: Human Immunodeficiency Virus; TB: Tuberculosis; ECG: electrocardiogram; SARS-CoV-2: Severe Acute Respiratory Syndrome Coronavirus 2; CDSS: clinical decision support system; EMR: electronic medical record: HRV: heart rate Variability; CXR; chest X-ray.
